# Fatty infiltration and cross-sectional area as indicators of muscle mass in osteoporosis: a meta-regression study

**DOI:** 10.3389/fendo.2025.1651505

**Published:** 2025-11-25

**Authors:** Genwen Sun, Yuee Dai, Liu Liu, Yu Du, Ping Jiang, Xiangkui Li, Chao Li, Tao Lin

**Affiliations:** 1 Sichuan Provincial Center for Mental Health, Sichuan Provincial People’s Hospital, School of Medicine, University of Electronic Science and Technology of China, Chengdu, China; 2 Key Laboratory of Psychosomatic Medicine, Chinese Academy of Medical Sciences, Chengdu, China; 3 Department of Pain, Sichuan Provincial People’s Hospital, School of Medicine, University of Electronic Science and Technology of China, Chengdu, China; 4 Sichuan Provincial People’s Hospital, School of Medicine, University of Electronic Science and Technology of China, Chengdu, China; 5 Faculty of Medicine, Autonomous University of Madrid, Madrid, Spain

**Keywords:** paraspinal muscles, multifidus, erector spinae, psoas major, psoas muscle, quadratus lumborum, iliopsoas, skeletal muscle

## Abstract

**Background:**

Paraspinal muscle morphology, including cross-sectional area (CSA) and fatty infiltration (FI), has been increasingly recognized as a potential imaging-based indicator of osteoporosis. However, the extent to which these muscle parameters differ across osteoporosis, osteopenia, and healthy populations remains unclear.

**Methods:**

A systematic meta-analysis was conducted based on 14 studies published from inception to January 25, 2025, comprising 125 effect size estimates related to CSA and FI across key paraspinal muscles. Pooled standardized mean differences (SMDs) were calculated using a random-effects model. Subgroup analyses were stratified by muscle group and diagnostic comparison. Three-level meta-regression models were implemented to examine the influence of study-level moderators, including age, sex, measurement level and comparison category.

**Results:**

A significant decrease in CSA was observed only in osteoporotic patients compared with controls, and multiple muscle groups were evaluated. In contrast, CSA differences in osteopenia were less consistent and appeared to vary by muscle type. FI demonstrated greater sensitivity across diagnostic comparisons, with significant increases observed in both osteopenia and osteo- porotic groups relative to controls, especially in the multifidus and erector spinae. Meta-regression identified age as a significant moderator, indicating that morphological differences diminish with increasing age. Both CSA and FI are associated with musculoskeletal deterioration in osteoporosis, with FI suggested to be relatively more sensitive and potentially capable of detecting early pathological changes during the osteopenia stage. However, when examined across specific measurement approaches for CSA and FI, the apparent advantage of FI was attenuated, and no clear difference in sensitivity was identified. The psoas major showed inconsistent findings across studies, likely due to its lower baseline fat content and lower responsiveness to aging.

**Conclusion:**

Both CSA and FI are associated with musculoskeletal deterioration in osteoporosis, with FI emerging as a more sensitive marker, potentially capable of detecting early pathological changes during the osteopenia stage. These findings highlight the value of paraspinal muscle assessments in osteoporosis research and clinical evaluation. Further studies are warranted to standardize measurement protocols and evaluate the integration of muscle morphology into imaging-based risk prediction models.

**Systematic Review Registration:**

https://www.crd.york.ac.uk/PROSPERO/view/CRD420251026322, identifier CRD420251026322.

## Introduction

Osteoporosis (OP) is a systemic skeletal disorder characterized by reduced bone mineral density (BMD) and deterioration of bone micro-architecture, which significantly increases the risk of fractures, particularly osteoporotic vertebral compression fractures (OVCFs) (1). A systematic review and meta-analysis based on standardized diagnostic criteria has shown that the prevalence of osteoporosis continues to rise with the global progression of population aging (2). The global prevalence of osteoporosis has reached 19.7%, exceeding 24.8% among individuals over the age of 60, while the prevalence of osteopenia is estimated at 40.4% (3). These figures indicate a likely future increase in the global burden of osteoporosis. Worldwide, osteoporosis is responsible for more than 8.9 million fractures annually, which corresponds to one fracture occurring approximately every three seconds (4). Low BMD is also strongly associated with spinal kyphosis (5), further contributing to both direct and indirect economic burdens on healthcare systems (2). Given its impact on quality of life and healthcare expenditures, early diagnosis and timely intervention are critical for the effective management of osteoporosis.

In recent years, increasing attention has been directed toward the role of paraspinal muscles in the pathophysiology of osteoporosis (1, 2, 6–10). This interplay has also been recognized as a central feature of osteosarcopenia, where concomitant bone and muscle loss synergistically contribute to fracture risk (11). The paraspinal muscles, which include the multifidus, erector spinae, and psoas major, are essential for maintaining spinal stability and providing mechanical loading to the vertebral column (12, 13). As force-generating structures, their contractions transmit mechanical stimuli to bone tissue, thereby promoting bone remodeling, facilitating micro-damage repair, and preventing the accumulation of structural defects (14). Degeneration of these muscles can lead to diminished strength and compromised mechanical loading, which may impair bone remodeling processes and hinder micro- damage repair, ultimately contributing to reduced BMD. Emerging evidence has demonstrated that paraspinal muscle degeneration, typically characterized by decreased cross-sectional area (CSA) and increased fatty infiltration (FI), is closely associated with osteoporosis (10, 15–17).

The interaction between muscles and bones involves not only biomechanical aspects but also com- plex molecular mechanisms. For example, the Wnt signaling pathway plays important roles in muscle- bone interactions. Wnt family members including Wnt3a, Wnt4 and Wnt10b regulate muscle and bone regeneration and differentiation through distinct mechanisms. The Wnt pathway promotes osteoblast differentiation and bone formation by activating *β*-catenin, while Wnt10b deficiency may be associated with increased muscle FI, potentially affecting BMD (18–20). In addition to Wnt signaling, several other muscle-derived signals have been shown to contribute simultaneously to the pathogenesis of osteoporosis and sarcopenia (21). For instance, irisin and L-*β*-aminoisobutyric acid (L-BAIBA) are two myokines shown to exert beneficial effects on bone metabolism (21). Irisin enhances osteogenesis by activating ERK/p38/AMPK pathways in osteoblasts (20), while L-BAIBA prevents mitochondrial damage induced by reactive oxygen species (ROS), thereby mitigating bone loss and preserving muscle function (22). Conversely, other myokines such as myostatin have deleterious effects; myostatin promotes osteoclastogenesis and bone resorption by activating the RANKL signaling pathway (23, 24).

Meanwhile, bone-derived hormones also play essential roles in this bidirectional muscle–bone crosstalk. Osteocalcin, a hormone secreted by osteocytes, has been reported to exhibit a negative correlation with bone mineral density and is known to function as a paracrine regulator of Wnt signaling (25, 26). In addition to its role in signaling, osteocalcin influences ATP utilization in osteoblasts. Sclerostin, another osteocyte-derived factor, further modulates these processes by suppressing Wnt signaling through inhibition of the SOST gene (27). These molecular mechanisms not only demonstrate the close muscle-bone relationship but also provide theoretical support for using paraspinal muscle morphological changes as assessment indicators for osteoporosis.

Previous studies have demonstrated an association between paraspinal muscle degeneration and reduced BMD, with some evidence suggesting that this relationship may be influenced by age, sex, and spinal level (28–30). Crawford et al. (2016) reported that both fat content and muscle volume in the lumbar paraspinal region vary by spinal level and increase with age, with fat infiltration being particularly pronounced at lower lumbar segments (30). Sollmann et al. (2020) reported that paraspinal muscle CSA showed no significant correlation with BMD at L2 level but demonstrated significant correlation at L4/5 level (28). Furthermore, paraspinal muscle degeneration may influence bone metabolism through inflammatory pathways (e.g., NF-*κ*B) and oxidative stress rather than solely via reductions in muscle mass or changes in FI (31). Some studies indicate that functional muscle CSA (excluding fat) correlates significantly with BMD, whereas total CSA (including fat) shows no clear correlation (32). These complexities and multi-factorial influences suggest that paraspinal muscle morphological changes as assessment and diagnostic indicators for osteoporosis require further research validation.

Based on this background, this meta-analysis aims to examine the correlation be-tween paraspinal muscle morphological changes and osteoporosis, evaluating their feasibility and clinical value as assessment indicators for osteoporosis. By integrating evidence from existing literature, this study will provide scientific support for applying paraspinal muscle morphological changes in osteoporosis assessment and diagnosis, while offering references for future re-search directions.

## Materials and methods

### Search strategy

This study was conducted in accordance with the Preferred Reporting Items for Systematic Reviews and Meta-Analyses (PRISMA) guidelines. Databases including PubMed, Embase, the Cochrane Library, and the Wanfang Database were searched from inception to 25 Jan 2025 (PROSPERO ID: CRD420251026322). Eligible studies compared paraspinal muscle morphological parameters between individuals with osteoporosis and those with normal BMD. The search strategy included combinations of the following keywords: “paraspinal muscles,” “multifidus,” “erector spinae,” “psoas major,” “Psoas Muscle,” “quadratus lumborum,” “iliopsoas,” “skeletal muscle,” “muscle density,” “cross-sectional area,” “fatty infiltration,” “muscle morphology,” “index,” “score,” “rating,” “quantitative analysis,” “bone mineral density,” “bone quality,” “osteoporosis,” “osteopenia,” “osteoporotic vertebral compression fractures,” and “fragility vertebral fracture.” The complete search syntax has been uploaded to the PROSPERO database.

### Inclusion and exclusion criteria

**Figure 1:**
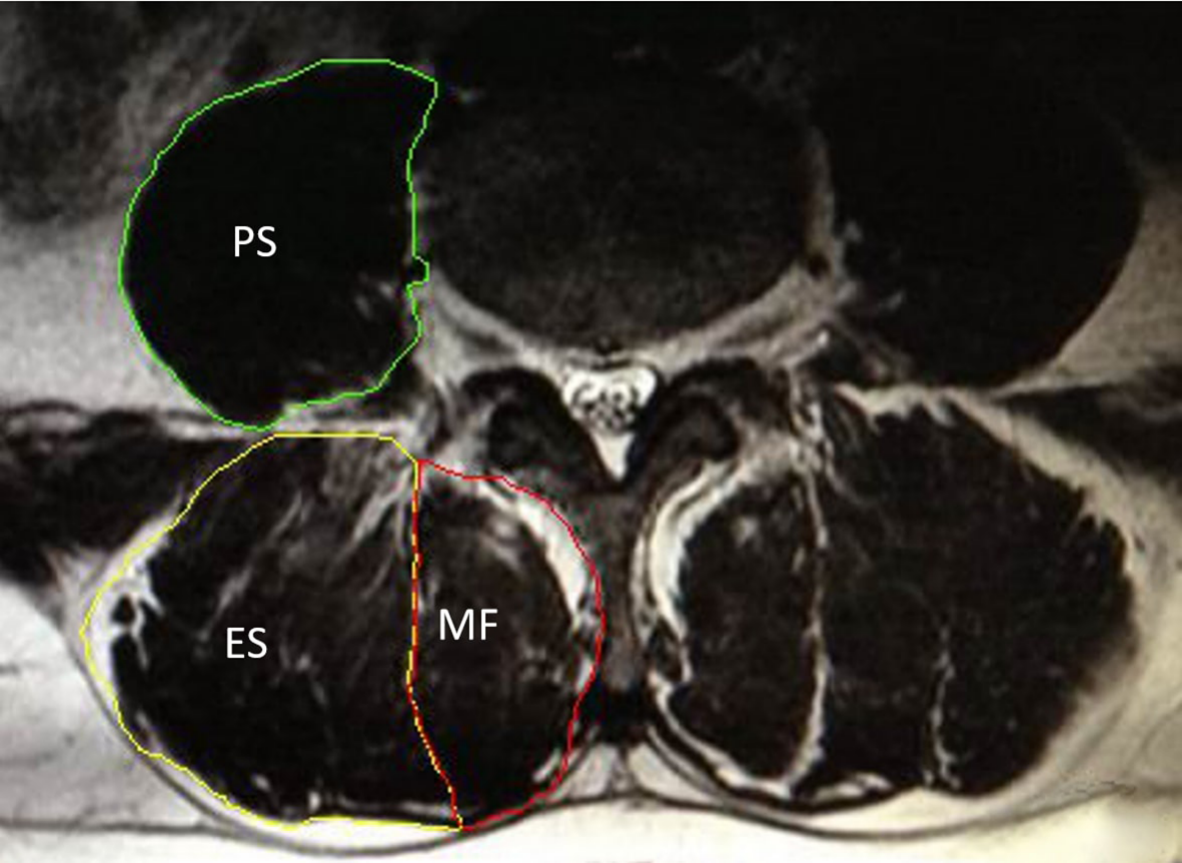
Axial MRI at the lumbar level showing segmentation of paraspinal muscles: psoas major (PS), erector spinae (ES), and multifidus (MF).

To be included in this meta-analysis, original studies had to meet the following criteria:(1) study types: including cross-sectional studies, cohort studies, case-control studies, and randomized controlled trials that provide original data on the correlation between paraspinal muscle morphometric measurements (such as CSA and FI) and osteoporosis;(2) study subjects: adults (*≥* 18 years) of any gender, including patients diagnosed with osteoporosis and healthy controls or those with normal BMD;(3) paraspinal muscle measurements: studies must report morphometric data of at least one paraspinal muscle (e.g., multifidus, erector spinae, psoas major, see **Figure 1**), primarily focusing on CSA and FI, as well as derived quantitative indices. For CSA, only studies using manual or semi-automated segmentation were included, given the limited accuracy and clinical validation of fully automated methods (33). Inter- observer and intra-observer reliability data were not required for inclusion, given that such metrics were often unreported in studies. (4) osteoporosis diagnosis: studies must specify diagnostic criteria for osteoporosis (e.g., based on BMD T-scores or quantitative computed tomography bone volume);(5) language: Chinese and English publications.

The exclusion criteria were:(1) study types: reviews, case reports, animal experiments, and basic scientific research;(2) incomplete data: studies lacking paraspinal muscle morphometric data or osteoporosis diagnostic data, or studies that cannot be included in pooled analysis due to missing data or inconsistent data presentation formats;(3) duplicate publications: overlapping data published by the same research team in different journals, with only the most recent or most complete study being included;(4) studies for which full text cannot be obtained.

### Data extraction and quality assessment

**Figure 2:**
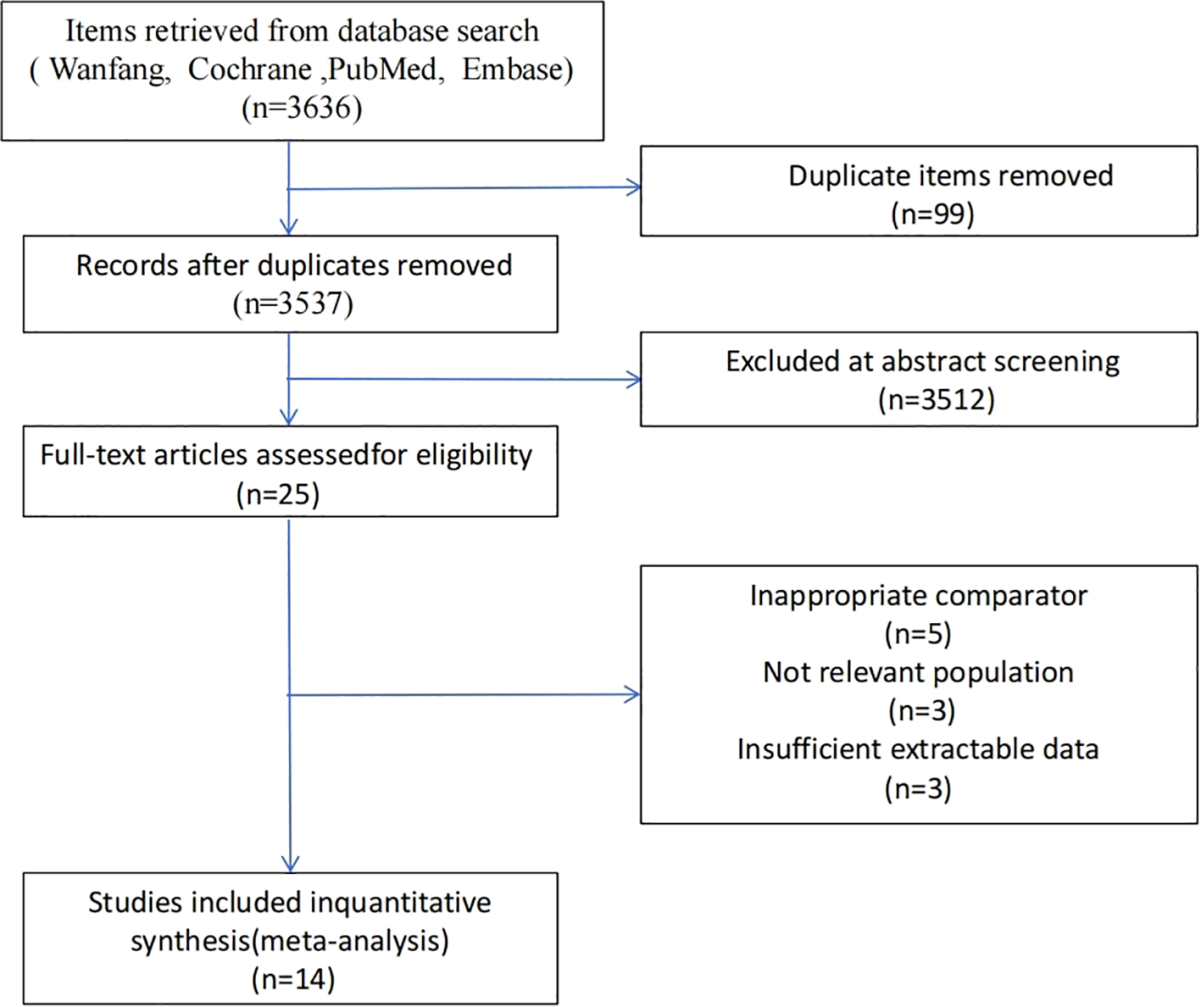
Selection process of the included studies.

Two investigators independently extracted the following information from each eligible study: first author’s name, year of publication, country of participant recruitment, study design, sample size, participants’ age, sex, body mass index (BMI), physical activity level, characteristics of case and control groups, equipment used for muscle and BMD measurements, segmentation techniques (e.g., manual and semi-automated), osteoporosis diagnostic criteria, and reported muscle morphological parameters. The extracted muscle parameters included CSA and FI of the posterior paraspinal muscles (multifidus, erector spinae and psoas major). The methodological quality of each included study was assessed using the standardized critical appraisal tools developed by the Joanna Briggs Institute (JBI) (34). Any discrepancies in data extraction or quality assessment were resolved through discussion with a third investigator. No study was rated as high risk of bias, all included studies were at low to moderate risk (see **Supplementary Table S2**). Following the screening process, fourteen studies met the inclusion criteria and were ultimately included in the analysis (see **Figure 2**).

### Statistical analysis

All effect sizes were calculated as standardized mean differences (SMDs) with corresponding 95% confidence intervals, based on means and standard deviations reported in the original studies. When necessary, values were harmonized to ensure consistency across studies (35). For CSA, bilateral averages were used when left and right sides were reported separately. For FI, various measurement techniques, including direct fat fraction estimation and proton density fat fraction (PDFF) calculations, were standardized and expressed as percentages. Pooled estimates were calculated using random-effects models with the DerSimonian–Laird method, as implemented in the *metafor* package in R (36, 37). Between-study heterogeneity was assessed using the *I*^2^ statistic and Cochran’s Q test. Subgroup analyses were conducted to examine effect sizes across different diagnostic group comparisons (e.g., osteoporosis vs. control, osteopenia vs. control) and across muscle types (multifidus, erector spinae, psoas major, and combined muscle groups).

To account for statistical dependency among effect sizes derived from the same study sample and to explore potential moderators, three-level meta-regression models were applied (36). These models included random intercepts at both the study and within-study levels to account for clustering. Moderator variables included outcome type (CSA vs. FI), diagnostic comparison group, mean age, proportion of female participants, BMI, vertebral measurement level (single vs. multiple), and muscle group. Model selection was guided by likelihood ratio tests and information criteria. Robust variance estimation with small-sample corrections was further applied to adjust for within-study clustering (36). All analyses were performed in R version 4.5.0 (38).

## Results

### Characteristics of the included studies

A total of 14 studies published within the past five years were included for the final analysis (6, 8–10, 16, 17, 39–46). All studies reported comparisons involving patients with osteoporosis and a control group, and most also included data on osteopenia, enabling additional com- parisons between osteopenia and either osteoporosis or control groups. Each study reported at least one of the two morphological indicators (CSA or/and FI), for at least one of the three key paraspinal muscle groups: multifidus, erector spinae, and psoas major. In addition, several studies provided aggregated data for multiple muscles that involved various paraspinal muscles, which were consistently categorized under a “multiple muscles” subgroup for analysis. Among the included studies, five re- ported both intra-observer and inter-observer reliability, providing corresponding intraclass correlation coefficients (ICCs). Except for Zhao et al. (2019), in which the inter-observer ICC for the psoas major was below 0.75, all other that reported ICC values indicated good or excellent reliability (see **Supplementary Table S2**). Most studies did not specify the ICC model used, but the methodological context implied the use of standard models such as ICC(2,1) or ICC(3,1).

Across all comparisons, a total of 125 unique datasets were extracted. The included studies varied in design: some recruited only healthy controls, while others focused on clinical populations with pre-existing spinal disease (excluding osteoporosis). Certain studies also adopted grouping strategies beyond the conventional osteoporosis versus control design, such as combining osteoporosis and osteopenia into a single experimental group or comparing osteoporosis against all non-osteoporotic individuals. These variations necessitated the standardization of group definitions prior to analysis.

All effect sizes were calculated as SMD, derived from the mean and standard deviation of the target variable (CSA or FI) reported in each study. For CSA, most values were obtained from manual segmentation of muscle regions (regions of interest) by radiologists, with measurements standardized to square millimeters. Some data were reported in alternative formats, such as means across multiple axial levels or normalized values (e.g., muscle index calculated as CSA divided by height squared) (40, 43). These formats were considered comparable and were included in the analysis. Han et al. (2022), for instance, reported both Relative Total Cross-sectional Area (rTCSA) and Relative Functional CSA (rFCSA), defined respectively as the total muscle area (including intramuscular fat and soft tissue) and the fat-free functional muscle area, each expressed as a ratio relative to the corresponding inter- vertebral disc area (8). Although reported as percentages, these values reflected CSA and were therefore also included in the analysis. When left and right sides were reported separately, the bilateral average was used. For FI, variability in measurement methodology was also noted. While some studies directly estimated the proportion of fat signal within the muscle, others used MRI-based techniques to calculate the PDFF (16). All FI outcomes were harmonized and expressed as percentages.

Pooled effect sizes were calculated using the *metafor* package in R, based on a random-effects model. Additionally, relevant study-level variables were extracted, including group-specific sample sizes, mean age, sex distribution, BMI of the samples, diagnostic criteria for osteoporosis and osteopenia, imaging modality (CT or MRI), and the vertebral level of measurement, in order to support subsequent meta- regression analyses, some variables have been included in **Supplementary Table S1** (see **Supplementary Materials**).

### Subgroup comparisons by muscle type and group category

**Figure 3:**
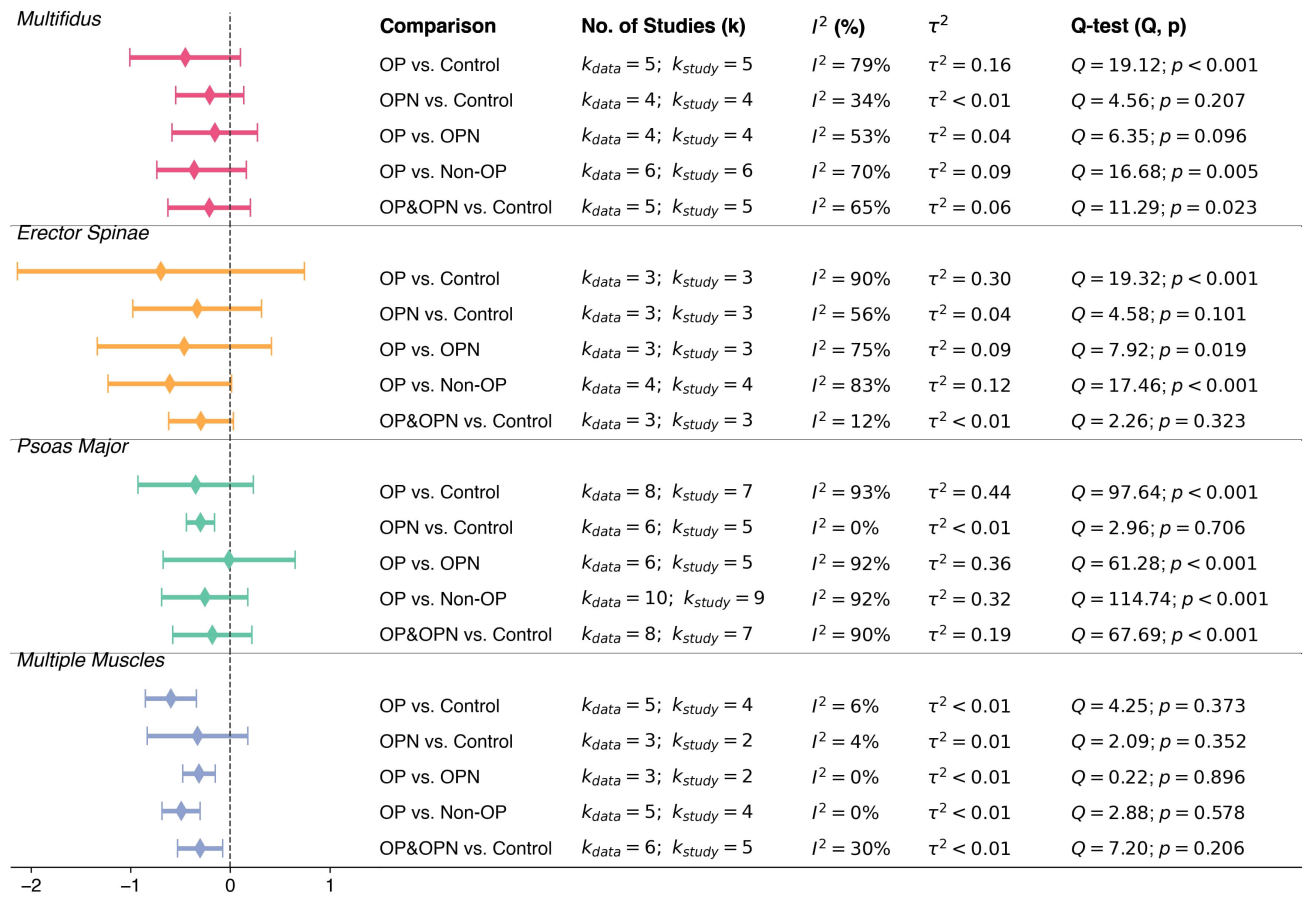
Summary of subgroup meta-analyses comparing cross-sectional area (CSA) across diagnostic groups by muscle type. Pooled standardized mean differences (SMDs) with corresponding 95% confidence intervals are displayed for comparisons between osteoporosis (OP), osteopenia (OPN), and control groups, stratified by muscle group (multifidus, erector spinae, psoas major, and multiple muscles). Heterogeneity measures (*I*^2^ and *τ*^2^) and Q-test results are also presented.

Since we collected the mean and standard deviation (SD) of CSA from all 14 included studies, we identified several variations in study design beyond the classic comparison of osteoporosis versus control. Some studies also included patients with osteopenia, while others compared combined osteoporosis and osteopenia groups with controls. To provide a comprehensive overview of CSA differences across these group categories, we conducted a series of stratified meta-analyses, grouping results by muscle type. Five types of comparisons were conducted: osteoporosis versus control (OP vs Control), osteopenia versus control (OPN vs Control), osteoporosis versus osteopenia (OP vs OPN), osteoporosis versus non-osteoporosis (OP vs Non-OP; including both OPN and controls), and combined osteoporosis and osteopenia versus control (OP&OPN vs Control; see **Figure 3**). For studies that did not clearly separate OP and OPN groups, we derived reconstructed comparisons where appropriate. For these types of comparisons, a negative SMD indicates a decrease in CSA in the left group compared to the right group.

The study and muscle specific results of each subgroup comparison are shown in **Supplementary Figures S1**-**S5**, with a comprehensive synthesis presented in **Figure 3**. Overall, the multiple muscles subgroup yielded consistently meaningful results in most comparisons, particularly in OP vs Control (SMD = *−*0.49, *I*^2^ = 0%) and OP&OPN vs Control (SMD = *−*0.30, *I*^2^ = 30%). However, in the comparison between osteopenia and control groups, the pooled effect did not reach statistical significance (SMD = *−*0.33, 95% CI: *−*0.83 to 0.18, *I*^2^ = 4.4%, see **Supplementary Figure S2**).

Of note, the number of contributing samples in the OPN vs Control and OP vs OPN comparisons for the multiple muscles subgroup was very limited, only three datasets derived from two studies, one of which (Tu et al., 2023) contributed two sex-specific samples (all-male and all-female) (43). Both comparisons showed no statistical heterogeneity (*I*^2^ = 0%) and non-significant Q-tests (*Q* = 2.09, *p* = 0.352 for OPN vs Control; *Q* = 0.22, *p* = 0.896 for OP vs OPN). Therefore, these results should be interpreted with caution due to limited statistical power.

Among the individual muscles analyzed, only psoas major showed a statistically significant reduction in CSA in the osteopenia group compared to controls (SMD = *−*0.30, 95% CI: *−*0.44 to *−*0.16, *I*^2^ = 0%). The apparent homogeneity should be interpreted with caution. This finding, however, supports the potential value of psoas major as an early imaging marker for muscle degradation in osteopenia, pending further confirmation in larger datasets.

**Figure 4:**
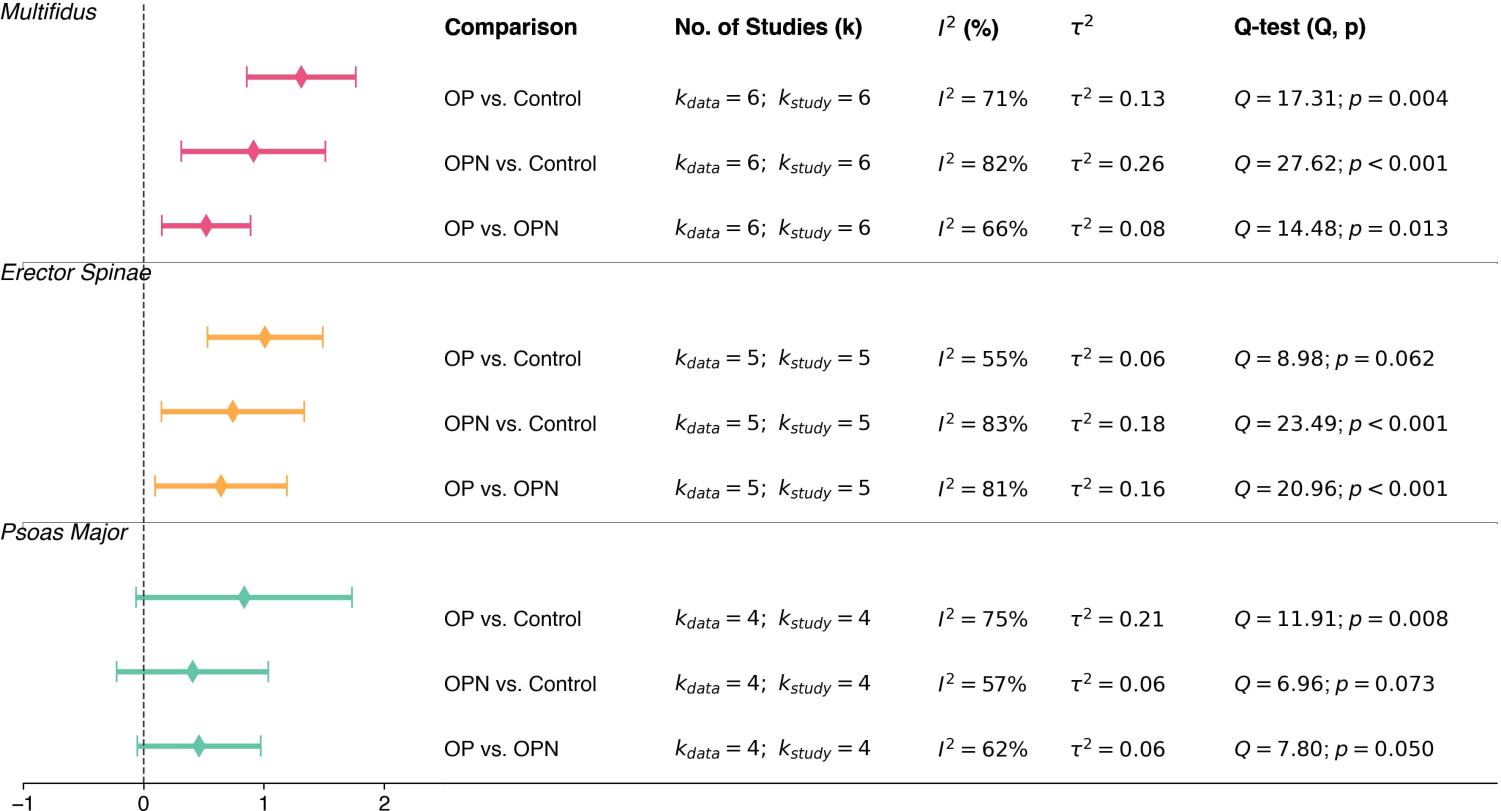
Summary of subgroup meta-analyses comparing fatty infiltration (FI) across diagnostic groups by muscle type. Pooled standardized mean differences with 95% confidence intervals are shown for comparisons between osteoporosis (OP), osteopenia (OPN), and control groups, stratified by multifidus, erector spinae, and psoas major muscles. Heterogeneity estimates (*I*^2^ and *τ*^2^) and Q-statistics are reported.

To comprehensively evaluate FI across different groups, we performed the same subgroup meta- analyses comparing osteoporosis versus control, osteopenia versus control, and osteoporosis versus osteopenia for each of the three primary paraspinal muscles: multifidus, erector spinae, and psoas major. These results are summarized in **Figure 4**, more details about the integrating pooled effect sizes and heterogeneity statistics showed from **Supplementary Figures S6**-**S8**. For all three types of comparisons, a positive SMD indicates that the FI of the left side group is higher than that of the right side group.

For the multifidus muscle, all three comparisons indicated significantly higher FI in osteoporotic or osteopenic individuals. The pooled SMDs were 1.31 (95% CI: 0.86 to 1.76, *I*^2^ = 71%, *p* = 0.004) for OP versus control, 0.91 (95% CI: 0.32 to 1.51, *I*^2^ = 82%, *p <* 0.001) for OPN versus control, and 0.52 (95% CI: 0.15 to 0.89, *I*^2^ = 66%, *p* = 0.013) for OP versus OPN. For the erector spinae, similar patterns were observed, with pooled SMDs of 1.01 (95% CI: 0.53 to 1.49, *I*^2^ = 55%, *p* = 0.062) for OP versus control, 0.74 (95% CI: 0.15 to 1.34, *I*^2^ = 83%, *p <* 0.001) for OPN versus control, and 0.64 (95% CI: 0.10 to 1.19, *I*^2^ = 81%, *p <* 0.001) for OP versus OPN. In contrast, psoas major did not show significant differences across the three comparisons (see **Figure 4**). The direction of effect was consistently positive, but none of the comparisons reached statistical significance for this muscle.

Across all comparisons in FI, most subgroups exhibited moderate to substantial heterogeneity, with *I*^2^ values exceeding 50 percent in nearly every analysis. In this variable, unlike the CSA, no comparison involving multiple muscle groups was conducted. This was due to the limited number of studies reporting FI data in the included studies, less than three datasets, rendering pooled analysis methodologically inappropriate.

### Three-level meta-regression analyses of study-level moderators

To account for dependencies among effect sizes derived from repeated samples, we implemented a three-level meta-regression using mixed-effects models. Our dataset included two outcomes (CSA and FI) from the same study, also individual studies often reported measurements for several muscles based on the same participant sample. Such a data structure unable to satisfy the independence assumption of conventional meta-analysis, as effect sizes within a study are inherently correlated, the subgroup analysis can only offer limited information. On the other hand, we also aimed to examine whether study-level characteristics, such as mean age, proportion of female participants, or measurement design (e.g., vertebral level selection), influenced the magnitude of CSA and FI effects. To address both the statistical correlation among effect sizes and the potential impact of these moderators, we adopted a hierarchical random-effects modeling strategy and fitted a series of multilevel models using the *metafor* package in R (36, 38).

In the hierarchical random-effects model, we introduced an additional clustering level (level 2) to account for within-study heterogeneity. Specifically, we modeled the study as a cluster, and included several moderators as fixed effects: the type of effect size (CSA or FI), the comparison category (e.g., OP vs Control, OPN vs Control), the mean age of participants, the proportion of female participants, the mean BMI, and whether the measurements were obtained from a single vertebral level or multiple levels of the vertebral column. Given that CSA and FI have distinct clinical directional interpretations, where a decrease in CSA indicates more severe osteoporosis, while an increase in FI similarly indicates musculoskeletal degradation. We applied a directional harmonization procedure to the CSA effect sizes to ensure that SMDs in the meta-regression analysis consistently indicated worse musculoskeletal status with higher values.

Model selection was guided by comparisons of alternative model specifications using the Akaike Information Criterion (AIC), the Bayesian Information Criterion (BIC), and likelihood ratio tests implemented via ANOVA. Two final models were retained for interpretation. Both models treated within-study variability and within-study variation as two levels of random intercepts. The results of the selected models are presented below.

We first evaluated a three-level meta-regression model using restricted maximum likelihood (REML) estimation, including study-level moderators such as mean age, female proportion, body mass index (BMI), outcome type (CSA or FI), vertebral level (single vs. multiple), and muscle group. All variance inflation factors (VIFs) for included moderators were below 2, indicating no multicollinearity.

Variance decomposition indicated that 88.9% of the total variability in effect sizes was attributable to heterogeneity, with 43.4% due to between-study differences and 45.4% due to within-study variability. Likelihood ratio testing further supported the inclusion of the third level, with the three-level model demonstrating a significantly better fit than a reduced two-level model without between-study variance (*χ*^2^ = 8.15, *p* = 0.004).

Among all moderators, only age and outcome types significantly predicted variation in effect sizes (see **Table 1**). Higher sample mean age was associated with smaller standardized mean differences (Estimate = *−*0.03, 95% CI *−*0.06 to *−*0.0005, *p* = 0.05), suggesting that group differences in CSA or FI may decrease with older age. Moreover, effect sizes derived from FI were significantly larger than those from CSA (Estimate = 0.36, 95% CI 0.17 to 0.54, *p <* 0.001), indicating that FI may be more sensitive to osteoporotic changes. The multiple muscles are close to statistical significance (Estimate = 0.37, 95% CI *−*0.02 to 0.76, *p* = 0.06); other moderators, including gender distribution, BMI, vertebral level, and other muscle groups, did not significantly explain the effect size variability.

**Table 1:** Results of the three-level meta-regression model evaluating the association between effect sizes and study-level moderators.

Moderator	Estimate (95% CI)	SE	z-value	p-value
Intercept	0.20 (*−*3.77; 4.16)	2.02	0.10	0.92
Age (year)	*−*0.03 (*−*0.06; *−*0.0005)	0.02	*−*1.99	0.05
Sample Female (%)	0.08 (*−*0.08; 0.24)	0.08	0.99	0.32
BMI (kg/m^2^)	0.01 (*−*0.15; 0.17)	0.08	0.14	0.89
Outcome Type (*FI)*	0.36 (0.17; 0.54)	0.09	3.80	*<* 0.001
Vertebral Level (*single*)	*−*0.05 (*−*0.60; 0.50)	0.28	*−*0.19	0.85
Muscles (Ref. *Erector Spinae*)				
*Multifidus*	*−*0.05 (*−*0.27; 0.17)	0.11	*−*0.42	0.68
*Psoas Major*	*−*0.12 (*−*0.34; 0.11)	0.11	*−*1.02	0.31
*Multiple Muscles*	0.37 (*−*0.02; 0.76)	0.20	1.87	0.06

Age represents the mean age of participants in each study. Sample Female (%) denotes the proportion of female participants, calculated per 10% increment. BMI refers to the average body mass index of the sample. Vertebral Level is a binary variable indicating whether muscle measurements were taken at a single vertebral level (coded as “single”) or across multiple levels (reference category). The moderator “Outcome Type (FI)” indicates whether the effect size was derived from fatty infiltration (FI) rather than cross-sectional area (CSA), with CSA used as the reference. Muscle group moderators compare *Multifidus*, *Psoas Major*, and *Mul- tiple Muscles* against the reference category. SE refers to the standard error of the estimated coefficient. All estimates are based on restricted maximum likelihood estimation (REML).

To ensure the robustness of moderator estimates under within-study dependence, we conducted a sensitivity analysis using cluster-robust variance estimation (CR2), specifying an assumed intra-cluster correlation of *ρ* = 0.7 (47). Under the robust specification, the effect of outcome type (FI vs CSA) remained marginally significant (Estimate = 0.37, 95% CI *−*0.04 to 0.78, *p* = 0.07), while all other moderators showed no statistically significant associations with effect sizes (see **Supplementary Table S4**).

To evaluate the independent contribution of diagnostic group comparisons, a second three-level meta-regression model was fitted, extending the first model by adding the comparison category (OP vs Control, OPN vs Control, etc.) as a categorical moderator. Among the moderators, the outcome type (FI vs CSA) remained a significant predictor of effect size magnitude (Estimate = 0.39, 95% CI 0.22 to 0.56, *p <* 0.001). In contrast to the previous model, none of the sample-level co-variates (age, female proportion, BMI) were significantly associated with effect sizes in this model (see **Supplementary Table S5**).

Notably, the comparison category showed a significant effect: effect sizes derived from studies comparing osteoporosis and controls (OP vs Control) were significantly larger than the reference group (Estimate = 0.49, 95% CI 0.27 to 0.70, *p <* 0.001). In contrast, effect sizes comparing osteopenia versus control (Estimate = 0.16, 95% CI *−*0.08 to 0.41) and osteoporosis versus non-osteoporosis (Estimate = 0.42, 95% CI *−*0.26 to 1.10) did not reach statistical significance (see **Supplementary Table S5**).

We also conducted a sensitivity analysis using cluster-robust variance estimation for this model. The intra-cluster correlation was set as *ρ* = 0.7 too. Under the robust specification, the effect of outcome type (FI vs CSA) remained statistically significant (Estimate = 0.41, 95% CI *−*0.008 to 0.83, *p* = 0.05), and effect sizes from comparisons between osteoporosis and control groups were significantly larger than the reference category (Estimate = 0.50, 95% CI 0.24 to 0.75, *p* = 0.005, see **Table 2**).

**Table 2:** Results of the three-level meta-regression model with cluster-robust standard errors assessing the influence of diagnostic group comparisons and study-level characteristics on effect sizes.

Moderator	Estimate (95% CI)	SE (df)	t-stat	p-value
Intercept	0.69 (*−*6.74 to 8.11)	3.03 (6.00)	0.23	0.83
Age (year)	*−*0.007 (*−*0.05 to 0.04)	0.02 (4.07)	*−*0.42	0.70
Sample Female (%)	0.001 (*−*0.24 to 0.24)	0.08 (3.73)	0.02	0.99
BMI (kg/m^2^)	*−*0.02 (*−*0.33 to 0.29)	0.13 (5.87)	*−*0.15	0.88
Outcome Type (*FI)*	0.41 (*−*0.008 to 0.83)	0.15 (3.69)	2.82	0.05
Vertebral Level (*single*)	*−*0.22 (*−*0.86 to 0.41)	0.24 (4.51)	*−*0.93	0.40
Muscles (Ref. *Erector Spinae*)				
*Multifidus*	*−*0.05 (*−*0.26 to 0.17)	0.08 (4.39)	*−*0.58	0.59
*Psoas Major*	*−*0.12 (*−*0.63 to 0.40)	0.19 (4.68)	*−*0.59	0.58
*Multiple Muscles*	0.25 (*−*0.35 to 0.85)	0.18 (2.76)	1.41	0.26
Comparison category (Ref. *OP vs. OPN)*				
*OP vs. Control*	0.50 (0.24 to 0.75)	0.10 (4.60)	5.06	0.005
*OPN vs. Control*	0.18 (*−*0.34 to 0.71)	0.21 (5.33)	0.89	0.41
*OP vs. Non-OP*	0.60 (*−*0.15 to 1.34)	0.24 (3.21)	2.46	0.09

Age represents the mean age of participants in each study. Sample Female (%) denotes the proportion of female participants, calculated per 10% increment. BMI refers to the average body mass index of the sample. Vertebral Level is a binary variable indicating whether muscle measurements were taken at a single vertebral level (coded as “single”) or across multiple levels (reference category). The moderator “Outcome Type (FI)” indicates whether the effect size was derived from fatty infiltration (FI) rather than cross-sectional area (CSA), with CSA used as the reference. Muscle group moderators compare *Multifidus*, *Psoas Major*, and *Multiple Muscles* against the reference category. Comparison Category indicates the type of diagnostic group contrast used in each effect size calculation. SE refers to the standard error of the estimated coefficient.

To examine whether measurement approaches contributed to heterogeneity, we performed a sensitivity analysis using cluster-robust variance estimation within a multilevel model, incorporating differences muscles, comparison categories, and measurement methods. For CSA, outcomes were classified as either raw area (reported in *mm*^2^ or averaged across slices) or normalized indices (CSA adjusted for height or other scaling factors). For FI, results were stratified by modality, including CT-based proxies or MRI-based measures. As shown in **Table 3**, the difference between the OP and control groups remained significant (Estimate = 0.46, 95% CI: 0.28–0.65, p *<* 0.001), while variation across measurement types did not materially affect the results (see **Table 3**).

**Table 3:** Results of the three-level meta-regression model with cluster-robust standard errors showing the effects of muscle group diagnostic comparison category and measurement type on effect sizes.

Moderator	Estimate (95% CI)	SE (df)	t-stat	p-value
Intercept	*−*0.06 (*−*0.73 to 0.61)	0.28 (7.01)	*−*0.20	0.85
Muscles (Ref. *Erector Spinae*)				
*Multifidus*	*−*0.04 (*−*0.25 to 0.18)	0.08 (4.32)	*−*0.47	0.66
*Psoas Major*	*−*0.18 (*−*0.71 to 0.36)	0.20 (4.31)	*−*0.89	0.42
*Multiple Muscles*	0.33 (*−*0.09 to 0.74)	0.15 (4.00)	2.20	0.09
Comparison category (Ref. *OP vs. OPN)*				
*OP vs. Control*	0.46 (0.28 to 0.65)	0.08 (5.99)	6.08	*<*0.001
*OPN vs. Control*	0.24 (*−*0.08 to 0.56)	0.13 (5.82)	1.85	0.12
*OP vs. Non-OP*	0.45 (*−*1.35 to 2.25)	0.28 (1.44)	1.60	0.30
Measurement (Ref. *Raw Area (CSA)*)				
*Relative Area (CSA)*	0.44 (*−*0.73 to 1.61)	0.40 (3.59)	1.09	0.34
*CT (FI)*	0.35 (*−*0.35 to 1.05)	0.16 (1.93)	2.24	0.16
*MRI (FI)*	0.84 (*−*2.22 to 3.90)	0.51 (1.50)	1.65	0.28

Muscle group moderators compare *Multifidus*, *Psoas Major*, and *Multiple Muscles* against the reference category. Comparison Category indicates the type of diagnostic group contrast used in each effect size calculation. SE refers to the standard error of the estimated coefficient.

To address potential sources of bias, we evaluated the impact of measurement reliability. Effect sizes did not differ according to whether ICCs were fully, partially, or not reported, indicating that measurement reliability was unlikely to bias the findings. In addition, we assessed potential publication bias. Visual inspection of both study-level and effect-level funnel plots (see **Supplementary Figure S9**) did not indicate pronounced asymmetry. Egger-type regression (48) including the standard error as moderator indicated borderline evidence of small-study effects (*β* = 1.90, p=0.054). Trim-and-fill analyses did not impute additional studies. Taken together, these findings suggest at most limited evidence for publication bias.

## Discussion

This meta-analysis systematically evaluated morphological alterations in paraspinal muscles, with a particular focus on CSA and FI, two measurements increasingly recognized as valuable imaging markers for osteoporosis (10, 15). Multiple group comparisons were performed among individuals with osteoporosis, osteopenia, and non-osteoporotic controls. Based on data from 14 studies and 125 effect sizes, the findings consistently demonstrated that osteoporosis is associated with decreased CSA and increased FI, reflecting quantifiable musculoskeletal deterioration.

Significant reductions in CSA were observed in the osteoporosis group compared with controls, particularly when multiple paraspinal muscle groups were assessed concurrently. In contrast, results from comparisons involving osteopenia were less consistent. Among individual muscles, only the psoas major showed a statistically significant reduction in CSA in osteopenia individuals relative to controls. This finding suggests that the psoas major may serve as a potential early imaging biomarker of muscle degeneration during the transition from normal bone density to osteopenia.

With respect to FI, inter-group differences were more pronounced. Individuals with osteoporosis exhibited significantly higher FI in both the multifidus and erector spinae muscles compared with osteopenia and control groups. However, no significant differences in FI were found in the psoas major across any diagnostic comparisons. These findings suggest that the susceptibility to fat infiltration may differ by muscle group. Notably, whether the psoas major should be classified as a paraspinal muscle remains a matter of debate in the literature, which may partially account for its divergent findings (12, 13).

Three-level meta-regression analyses further indicated that effect sizes based on FI were significantly greater than those based on CSA, suggesting that FI may be a more sensitive marker of osteoporotic changes. Among the diagnostic contrasts, the largest effect sizes were observed in the comparison between osteoporosis and control groups. Comparisons involving osteopenia alone generally did not yield significant differences, indicating that muscle alterations in this group may be more localized or less pronounced.

### Association between paraspinal muscle CSA and osteoporosis

Paraspinal muscles are important stabilizers for maintaining the dynamic balance of the spine, and are closely related to the vertebral load distribution in terms of morphology and function (12, 13). During muscle contraction, mechanical forces are transmitted to vertebral bone tissue through biomechanical leverage. Osteocyte located within the hydroxy-apatite mineral matrix are able to sense these mechanical strains and coordinate the activities of osteoclast and osteoblasts to promote a continuous bone remodeling process (14, 49).

Our analysis revealed a significant association between CSA and osteoporosis. In patients with osteoporosis, the reduction in CSA in multiple paraspinal muscles appears to be more pronounced, although considerable variability may exist between individuals. From a mechanistic perspective, muscle atrophy may influence bone metabolism through three principal pathways. First, a reduction in muscle mass directly decreases the dynamic mechanical load applied to the vertebrae. Second, impaired spinal stability may lead to abnormal stress distribution along the vertebral column, which accelerates the accumulation of micro-damage. Third, patients with osteoporosis often experience progressive muscle loss due to chronic pain or reduced mobility, resulting in a self-reinforcing degenerative cycle (50, 51).

In the subgroup analysis, we did not observe significant differences at the level of individual muscles. This is consistent with previous studies that have reported inconsistent findings and have suggested that factors such as age, sex, and muscle location may exert important influence (28, 52). In our study, we examined these potential effects, and in one of our models, age emerged as a significant moderator. As age increased, the differences in muscle morphological indicators, whether CSA reduction or FI increase, between diagnostic groups became less apparent. This finding confirms the moderating role of age and also suggests that, in older populations, age-related degeneration and muscle atrophy may reduce the sensitivity of muscle-based morphological measurements in identifying osteoporosis-related changes.

The results of meta-regression also support the progressive degeneration hypothesis, which means that muscle morphological deterioration occurs simultaneously with a decrease in bone mineral density, progressing from a normal bone state to osteopenia and finally to osteoporosis. This trend is particularly evident in FI, which demonstrated a clearer and more consistent pattern across diagnostic stages compared to CSA. As illustrated in **Figures 3** and **4**, FI may serve as a more sensitive indicator of muscle degeneration related to bone health. In contrast, CSA changes during the osteopenia stage appear to be more heterogeneous and may require more refined imaging techniques to capture subtle morphological alterations.

### Association between paraspinal muscle FI and osteoporosis

The association between paraspinal muscle fatty infiltration and osteoporosis may be mediated through multiple pathways. From a biomechanical perspective, the replacement of muscle fibers with adipose tissue directly impairs contractile function, resulting in a reduction of mechanical loading on the bone (14, 49). This alteration disrupts osteocyte-mediated mechanotransduction signaling, thereby promoting the migration and differentiation of osteoclast precursors, which may accelerate bone resorption (14). This process occurs in parallel with the reduction in paraspinal muscle cross-sectional area, and together these pathological changes compromise spinal dynamic stability and increase the risk of abnormal stress distribution within the vertebral bodies.

From a metabolic and inflammatory perspective, infiltrated adipose tissue releases pro-inflammatory cytokines such as tumor necrosis factor alpha (TNF-*α*) and adipokines such as serum resisting through paracrine signaling mechanisms. TNF-*α* suppresses osteoblast function at specific stages of differentiation while simultaneously promoting the proliferation and differentiation of osteoclast (53). Serum resisting has also been shown to directly enhance osteoclastogenesis (54). These factors, in combination with sclerostin up-regulation resulting from reduced mechanical loading, contribute to a converging pathological process that promotes bone degradation in osteoporosis.

As previously discussed, FI appears to be more sensitive than CSA in detecting musculoskeletal changes. In the subgroup analyses, FI of several muscles, particularly the multifidus and erector spinae, showed statistically significant differences across all group comparisons. This suggests that these two muscles exhibit distinct morphological differences in osteoporotic patients, osteopenic patients, and non-osteoporosis controls. Consistently, the meta-regression results also indicated that FI demonstrates higher diagnostic sensitivity for osteoporosis than CSA.

When further exploring whether specific measurement approaches within FI and CSA yielded differential sensitivity, no meaningful differences were detected. This may be due to the relatively small number of included studies, with stratified analyses under each measurement method further reducing statistical power. Moreover, the differences between measurement methods in the current dataset may not have been sufficiently large to produce detectable variations in effect sizes. In addition, our use of a conservative analytic strategy may have limited the ability to capture subtle between-method differences. Together, these considerations suggest that the significant results observed for FI and CSA should be interpreted with appropriate caution.

Cross-sectional area serves as a direct measure of muscle size, and larger CSA is generally associated with greater muscle strength (55, 56). In contrast, indices reflecting intramuscular adipose tissue, such as FI, are considered indicators of muscle quality (57). Sarcopenia and osteopenia frequently coexist in the aging population, and FI may serve as a potential biomarker not only for osteopenia but also for osteosarcopenia (58). Fat infiltration increases at an early stage of BMD loss and affects bone metabolism through metabolic pathways, thus promoting bone degradation in osteoporosis, whereas a decrease in CSA may become evident only after progression to osteoporosis (14, 46, 54). This hypothesis is supported by previous studies reporting that FI may serve as an earlier marker of muscle degeneration than CSA (8, 29, 59). A study has clearly pointed out that there is a clear correlation between FI but not CSA and bone mineral density at the lumbar spine (29). MRI and CT imaging studies have shown that individuals with low BMD consistently exhibit higher levels of FI in paraspinal muscles, even in the absence of marked reductions in muscle CSA (8, 40). Also, muscle quality can be improved independently of muscle size through resistance training interventions (60). Several meta- analyses have demonstrated that dynamic and progressive resistance training can lead to small but significant improvements in bone mineral density at clinically relevant sites, including the lumbar spine, femoral neck, and total hip (61–63). These findings suggest that in the stage of osteopenia, an increase in FI may occur without a marked reduction in CSA, potentially indicating a risk of coexisting sarcopenia. At this stage, resistance training may improve muscle quality, thereby reducing the risk of both sarcopenia and osteopenia, and potentially slowing or even reversing the progression toward osteoporosis.

Previous studies have reported that FI in the psoas major shows significant differences in individuals with osteoporosis (16, 42). Other research has suggested that reduced fat infiltration in the psoas muscle may predict the presence of osteoporosis and increase the risk of bone fractures in young and middle- aged populations (64). However, in our meta-analysis, such differences were not consistently significant. This inconsistency may be attributed to the fact that the psoas major typically exhibits much lower levels of fat infiltration compared to the multifidus and erector spinae muscles, also, its fat content does not appear to vary significantly with age, sex, or lumbar level in healthy or asymptomatic individuals (65–67).

In summary, both CSA and FI represent clinically relevant morphological markers for assessing musculoskeletal changes in osteoporosis. CSA is widely used as an anthropometric indicator due to its high accessibility and reproducibility in both clinical and research contexts (68). Both CT and MRI have demonstrated excellent accuracy in quantifying CSA and FI, with measurements that are strongly correlated and largely interchangeable. However, FI may provide additional insight into muscle quality and degeneration that may not be captured by CSA alone (55).Despite its potential advantages, the consistency of FI measurements across studies remains limited by methodological heterogeneity, including variations in MRI protocols, fat quantification algorithms, and anatomical levels assessed (69–71).

### Study limitations and future directions

This study has several notable limitations. Most included studies involved clinical or degenerative cohorts and were predominantly conducted in Asian populations, which may limit the generalizability of our findings. The analysis included data from only 14 studies. Although methodological efforts were made to reduce the impact of limited study inclusion on interpretability, the relatively small number of studies remains a constraint. In subgroup analyses, some comparisons exhibited extremely low heterogeneity, with I² values close to zero. Upon inspection, no duplicated samples were identified, which may suggest consistency among study populations. However, given the limited number of included studies, particularly within subgroups, there remains a risk that true heterogeneity may have gone undetected due to insufficient statistical power.

Bone mineral density in Asian older adults, particularly at lower lumbar levels, tends to decline more rapidly than in Caucasians, which may partly explain the geographic concentration of studies in Asia. In contrast, BMD decline in Caucasians often follows a mixed pattern (66). Given Caucasians generally higher physical activity levels, muscle quality deterioration may precede muscle mass loss during the progression from osteopenia to osteoporosis in a more universally applicable manner (72, 73). Future studies including multi-ethnic populations and incorporating physical activity and muscle function assessments are needed to validate this conclusion. The lack of consistent reporting on inter- observer and intra-observer reliability across the included studies prevented us from incorporating this factor into the inclusion criteria, potentially contributing to unquantified measurement variability.

Although we collected several study-level variables and examined them as potential mediators in meta-regression analyses, certain variables could not be fully addressed. For example, while the vertebral levels at which measurements were taken ranged from L1 to S1 across studies, we were only able to categorize them as either single-level or multi-level assessments due to the limited number of studies available. Furthermore, we did not include imaging modality (CT vs. MRI) in the subgroup analyses or meta-regression modeling, as only two of the included studies employed CT to assess CSA or FI. This limitation rendered modality-based analyses unfeasible and may have affected the robustness of our modeling approach. Although we initially attempted to include vertebral level and measurement technique as moderators in the meta-regression model, these variables were ultimately excluded due to insufficient and highly unbalanced data. For instance, the vertebral level measurements varied widely from L1 to S1, with many studies reporting averaged values across multiple levels. These issues made it difficult to construct a stable model without overfitting. We acknowledge this limitation and emphasize the need for more standardized and detailed reporting in future studies.

Although FI appeared to demonstrate a stronger association with osteoporosis than CSA, the number of studies reporting FI measurements was relatively small. This raises the possibility of publication bias, as studies with significant findings are more likely to be published. Therefore, the apparent superiority of FI should be interpreted with caution, and further well-designed studies are needed to confirm these findings.

Overall, the present study supports the use of paraspinal muscle CSA and FI as morphological indicators of osteoporosis, with FI demonstrating particular promise. FI may have the potential to indicate the risk of osteoporosis and fracture even during the osteopenia stage. However, the integration of these two parameters into clinical risk prediction models remains limited. Although several studies have attempted to incorporate such indicators (39, 40), issues such as high false-positive rates and low area under the curve (AUC) values have restricted their clinical applicability. Moreover, CSA has also been associated with other spinal conditions, including lumbar disc herniation and spinal stenosis, which further complicates its interpretability in the context of osteoporosis (74, 75). Moreover, we were unable to fully separate cohorts with “pure” osteoporosis or osteopenia from those with concurrent degenerative conditions. Even when not explicitly reported, comorbid degenerative changes are likely to be present in clinical cohorts of older adults with osteoporosis (76). As such, distinguishing truly asymptomatic or healthy samples from clinical degenerative cohorts was not feasible with the available data. This limitation may affect the generalizability of our findings, and highlights the need for future studies with clearer characterization of comorbidities.

Therefore, additional studies are needed to clarify the influence of various factors, including age, ethnicity, imaging modality, and vertebral level of measurement. A standardized protocol for the estimation of muscle morphological parameters should be established before these indicators can be reliably incorporated into clinically applicable imaging-based risk stratification models.

## Conclusion

This meta-analysis provides comprehensive evidence supporting the role of paraspinal muscle morphology, particularly CSA and FI in the evaluation of osteoporosis. Among the two indicators, FI demonstrated greater sensitivity in distinguishing between diagnostic groups, with consistent differences observed even in the osteopenic stage. However, the differences were no longer significant when we examined the specific measurement approaches used to calculate CSA and FI. Neither the methods for FI nor those for CSA demonstrated notable sensitivity. Therefore, the relative advantage of FI over CSA requires further investigation at the level of measurement methodology before it can be firmly established. CSA reduction, although evident in patients with osteoporosis, showed greater heterogeneity and was less sensitive in early disease stages. Meta-regression further identified age as a significant moderator, suggesting that age-related musculoskeletal degeneration may obscure group-level differences, particularly in older populations. While both CSA and FI hold potential as imaging-based markers for musculoskeletal deterioration associated with osteoporosis, their integration into clinical risk stratification models requires further validation. Future research should aim to standardize measurement protocols and address confounding factors such as age, ethnicity, imaging modality, and vertebral level to enhance the clinical utility of these morphological parameters.

## Data Availability

The raw data supporting the conclusions of this article will be made available by the authors, without undue reservation.
