# Supplementary material for: Fatty infiltration and cross-sectional area as indicators of muscle mass in osteoporosis: a meta-regression study
Source: Front Endocrinol (Lausanne). 2025 Nov 25;16:1651505. doi: 10.3389/fendo.2025.1651505 (PMC12685714; doi:10.3389/fendo.2025.1651505)
Supplement: Supplementary file 1 [file DataSheet1.pdf]

Supplementary Materials: Fatty Infiltration and Cross-Sectional Area as Indicators of Muscle Mass in Osteoporosis: A Meta-Regression Study

Genwen Sun[1,2], Yuee Dai[1], Liu Liu[1], Yu Du[1], Ping Jiang[1], Xiangkui Li[1], Chao Li[3], Tao Lin[1,*]

[1] Department of Pain, Sichuan Provincial People's Hospital, School of Medicine, University of Electronic Science and Technology of China, Chengdu 610031, China.
[2] Sichuan Provincial Center for Mental Health, Sichuan Provincial People's Hospital, School of Medicine, University of Electronic Science and Technology of China, Chengdu 610031, China.
[3] Faculty of Medicine, Autonomous University of Madrid, Madrid, 28029, Spain.

Emails: sungen-wen@med.uestc.edu.cn (G.S.); daiyuee@med.uestc.edu.cn (Y.D.); liuliu@med.uestc.edu.cn (L.L.); duyu@med.uestc.edu.cn (Y.D.); 331895554@qq.com (P.J.); 1727415483@qq.com (X.L.); ericlimed@gmail.com (C.L.); 65309706@qq.com (T.L.)

[*]Correspondence: 65309706@qq.com

Table S1: Basic characteristics of included studies.

Study	Design	Country	Muscle	Outcome	Measure	Criteria	Comparison	Level	Recruitment
Deng(2024) [1]	Cross-sectional	China	PM; MM	CSA	MRI	MRI+ADC	OP; CONTROL	L3	Clinical
Gassert(2022) [2]	Cohort Study	Germany	MM	PDFF(FI); AverCSA	MRI	QCT	OP/OPN; CONTROL	L1; L2; L3	Clinical
Han(2022) [3]	Case-control	China	MF; ES; PM	rTCSA; FI; rFCSA	MRI	DXA	OP; OPN; CONTROL	L4; L5; S1	Clinical (lumbar spinal stenosis)
Huang(2022) [4]	Cross-sectional	China	PM; MM	MI (CSA)	CT	DXA	OP; CONTROL	L3	Clinical
Li(2022) [5]	Cross-sectional	China	MF; ES; PM	FF(FI); AverCSA	CT	QCT	OP; OPN; CONTROL	L3; L4; L5; S1	Community
Li(2023) [6]	Cross-sectional	China	MF	CSA	CT	DXA	OP; CONTROL	L3	Clinical
Li(2024) [7]	Cross-sectional	China	MF; ES; PM	AverCSA; PDFF(FI)	MRI	QCT	OP; OPN; CONTROL	L3; L4; L5; S1	Clinical
Wang(2024) [8]	Cross-sectional	China	MM	AverCSA; DFF(FI)	MRI	DXA	OP; OPN; CONTROL	L1; L2; L3; L4; L5	Clinical
Xiang(2023) [9]	Cross-sectional	China	MF; ES; PM	FC(FI)	CT	QCT	OP; OPN; CONTROL	L3	Mixed
Zhao(2019) [10]	Cross-sectional	China	MF; ES; PM	PDFF(FI)	MRI	QCT	OP; OPN; CONTROL	L2; L3; L4; L5	Community
Tu(2023) [11]	Cross-sectional	China	MM; PM	MI(CSA)	CT	QCT	OP; OPN; CONTROL	L3	Mixed
Zhang(2022) [12]	Cross-sectional	China	PM	MI(CSA)	CT	DXA	OP; Non-OP	L3	Clinical
Zhang(2021) [13]	Case-control	China	MF; ES; PM	CSA	MRI	DXA	OP; Non-OP	L3; L4; L5	Clinical

Continued on next page

Table S1 Continued: List of All Included Studies

Study	Design	Country	Muscle	Outcome	Measure	Criteria	Comparison	Level	Recruitment
Lee& Bae(2023) [14]	Case-control	South Korea	MF; PM	MI(CSA); CSA; FI	MRI	DXA	OP; OPN; CONTROL	L3; L4; L5; S1	Mixed

Note: Muscle includes MM (multiple muscles), MF (multifidus), ES (erector spinae), and PM (psoas major). Outcome types include MI (muscle index, calculated as $CSA/height^2$), CSA (cross-sectional area), FC (fat content), PDFF (proton density fat fraction), FI (fatty infiltration), AverCSA (average CSA across lumbar levels), rTCSA (relative total cross-sectional area), rFCSA (relative functional cross-sectional area), and DFF (degree of fat infiltration). Osteoporosis diagnostic methods include DXA (dual-energy X-ray absorptiometry), QCT (quantitative computed tomography), and ADC (apparent diffusion coefficient). Group labels include OP (osteoporosis), OPN (osteopenia) and control. Recruitment sources are categorized as Clinical (patients from spine or orthopedic departments), Mixed (hospital outpatients or combined sources), and Community (participants recruited from the general population).

**Author and Year**	**Study Type**	**Q1**	**Q2**	**Q3**	**Q4**	**Q5**	**Q6**	**Q7**	**Q8**	**Q9**	**Q10**	**Q11**	**Risk of Bias**
Gassert(2022) [2]	Prospective Observational	Yes	Yes	Yes	Yes	Yes	Yes	Yes	No	Yes	Not applicable	Yes	Low
Han et al. (2022) [3]	Case-control	Yes	Yes	Yes	Yes	Yes	No	No	Yes	Unclear	Yes	/	Low
Lee& Bae(2023) [14]	Case-control	Yes	Yes	Yes	Yes	Yes	No	No	Yes	Unclear	Yes	/	Low
Zhang et al. (2021) [13]	Case-control	Yes	Yes	Unclear	Yes	Yes	Yes	Yes	Yes	Not applicable	Yes	/	Moderate
Deng et al. (2024) [1]	Cross-sectional	Yes	Yes	Yes	Yes	No	No	Yes	Yes	/	/	/	Low
Huang et al. (2022) [4]	Cross-sectional	Yes	Yes	Yes	Yes	No	No	Yes	Yes	/	/	/	Low
Li et al. (2022) [5]	Cross-sectional	Yes	Yes	Yes	Yes	Yes	Yes	Yes	Yes	/	/	/	Low
Li et al. (2023) [6]	Cross-sectional	Yes	Yes	Yes	Yes	No	Yes	Unclear	Yes	/	/	/	Low
Li et al. (2024) [7]	Cross-sectional	Yes	Yes	Yes	Yes	Yes	Yes	Yes	Yes	/	/	/	Low
Wang et al. (2024) [8]	Cross-sectional	Yes	Yes	Yes	Yes	Yes	Yes	Yes	Yes	/	/	/	Low
Xiang et al. (2023) [9]	Cross-sectional	Yes	Yes	Yes	Yes	No	No	Yes	Yes	/	/	/	Low
Zhao et al. (2019) [10]	Cross-sectional	Yes	Yes	Yes	Yes	Yes	Yes	Yes	Yes	/	/	/	Low
Tu et al. (2023) [11]	Cross-sectional	Yes	Yes	Yes	Yes	No	No	Yes	Yes	/	/	/	Moderate
Zhang et al. (2022) [12]	Cross-sectional	Yes	Yes	Yes	Yes	No	Unclear	Yes	Yes	/	/	/	Moderate

Assessed using the JBI Checklist for Cohort study [15], for Case-Control Studies [16] and Cross- sectional studies [17].

Table S2: Methodological Quality Assessment for Studies Using the JBI Checklist

**Author and Year**	**Intra-observer**	**Inter-observer**	**Notes**
Deng et al. (2024) [1]	No	Yes	All ICC values are ≥ 0.75, indicating good measurement consistency. Authors reported ICC values but did not specify the exact ICC model.
Gassert et al. (2022) [2]	Yes	No	All ICC values are ≥ 0.75, indicating good measurement consistency. Authors reported ICC values but did not specify the exact ICC model.
Han et al. (2022) [3]	Yes	Yes	All ICC values are ≥ 0.75, indicating good measurement consistency. ICC(2,1) model used.
Huang et al. (2022) [4]	No	No	ICC not reported.
Li et al. (2022) [5]	No	No	ICC not reported.
Li et al. (2023) [6]	No	No	ICC not reported.
Li et al. (2024) [7]	Yes	No	ICC > 0.80 (excellent); ICC reported without specifying model.
Tu et al. (2023) [11]	Yes	Yes	All ICC values are ≥ 0.75, indicating good measurement consistency. ICC(2,1) model used.
Wang et al. (2024) [8]	Yes	Yes	All ICC values are ≥ 0.75, indicating good measurement consistency. ICC(3,1) model used.
Xiang et al. (2023) [9]	No	No	ICC not reported.
Zhang et al. (2021) [13]	Yes	Yes	All ICC values are ≥ 0.75, indicating good measurement consistency. ICC(3,1) model used.
Zhao et al. (2019) [10]	Yes	Yes	Intra-observer: All ICC values are ≥ 0.75, indicating good measurement consistency. Inter-observer: psoas major = 0.67 (moderate); Erector spinae and multifidus ≥ 0.85 (excellent). ICC(2,1) model used.
Zhang et al. (2022) [12]	No	No	ICC not reported.
Lee & Bae (2023) [14]	No	No	ICC not reported.

Table S3: Summary of Observer Reliability and ICC Values Reported in Included Studies

Subgroup Comparisons by Muscle Type and Group Category

Figure S1 Osteoporosis vs. Control in CSA

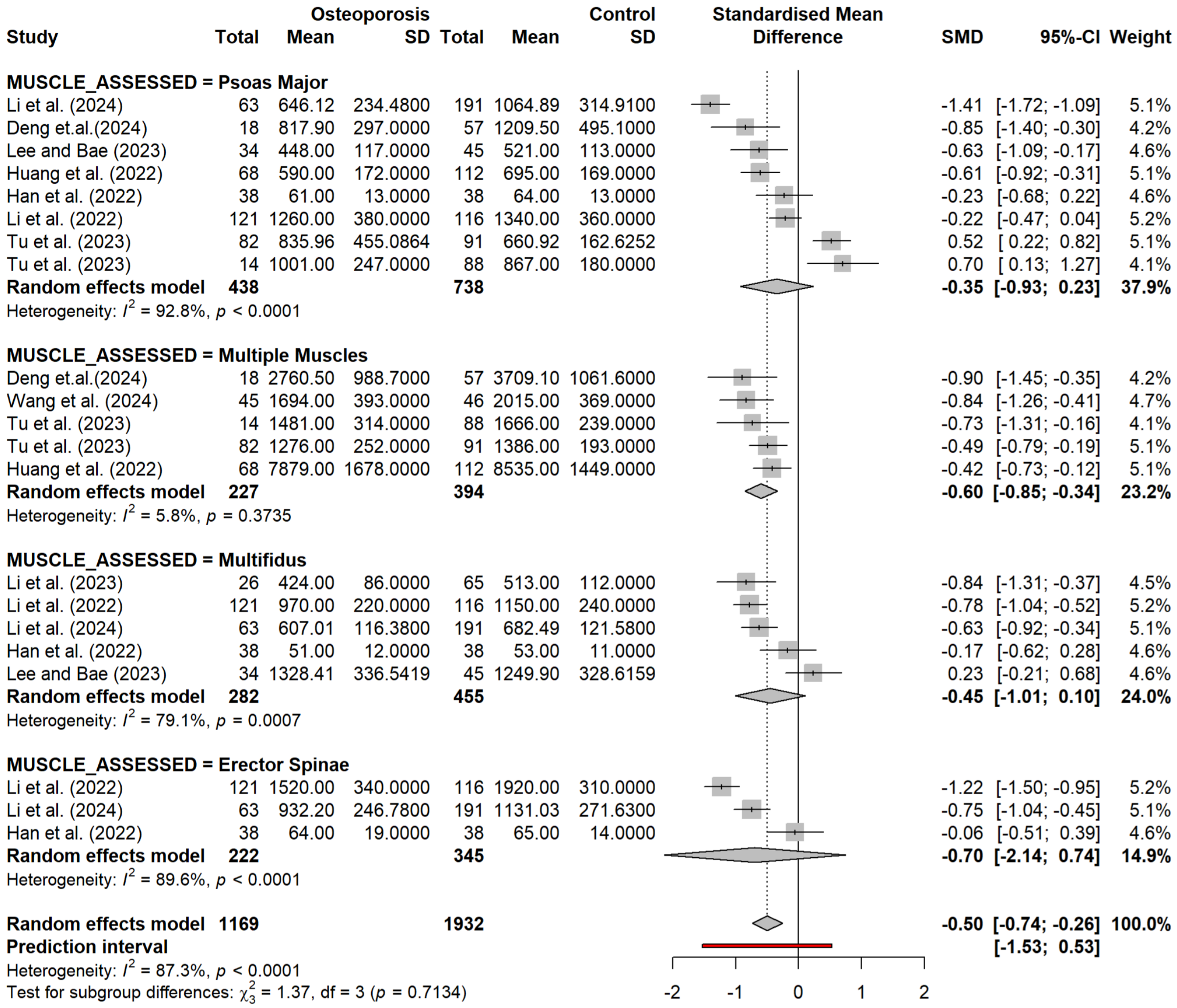

Figure S1: Forest plot of the standardized mean differences in cross-sectional area between individuals with osteoporosis and controls, categorized by different muscle groups.

Figure **S1** presents the forest plot of the standardized mean differences (SMD) in cross-sectional area (CSA) between patients with **osteoporosis** and **control** groups. Studies were categorized according to the specific muscle assessed: psoas major, multifidus, and erector spinae. Some studies also reported CSA values for multiple muscles, including at least one paraspinal muscle, and these were analyzed as a separate subgroup in the current meta-analysis.

The pooled effect size for the psoas major group (5 effect sizes) indicated a non-significant reduction in CSA among osteoporosis patients compared to controls (SMD $= -0.35$, 95% CI: -0.93 to 0.23, $I^2 = 92.8\%$). Similarly, the pooled estimates for the erector spinae and multifidus groups were also non-significant (SMD $=$ -0.70, 95% CI: -2.14 to 0.74, $I^2 = 89.6\%$ for the erector spinae group; SMD $= -0.45$, 95% CI: -1.01 to 0.10, $I^2 = 79.1\%$ for the multifidus group). Only the multiple muscles group showed a statistically significant reduction in CSA (SMD $= -0.60$, 95% CI: -0.85 to -0.34, $I^2 = 5.8\%$).

Overall, the random-effects model revealed a moderate reduction in CSA in the osteoporosis group

compared to controls (SMD = −0.50, 95% CI: −0.74 to −0.26), with substantial heterogeneity observed (I^2 = 87.3%). However, this analysis includes repeated studies assessing different muscles, the overall findings should be further examined using mixed-effects meta-regression models.

Figure S2 Osteopenia vs. Control in CSA

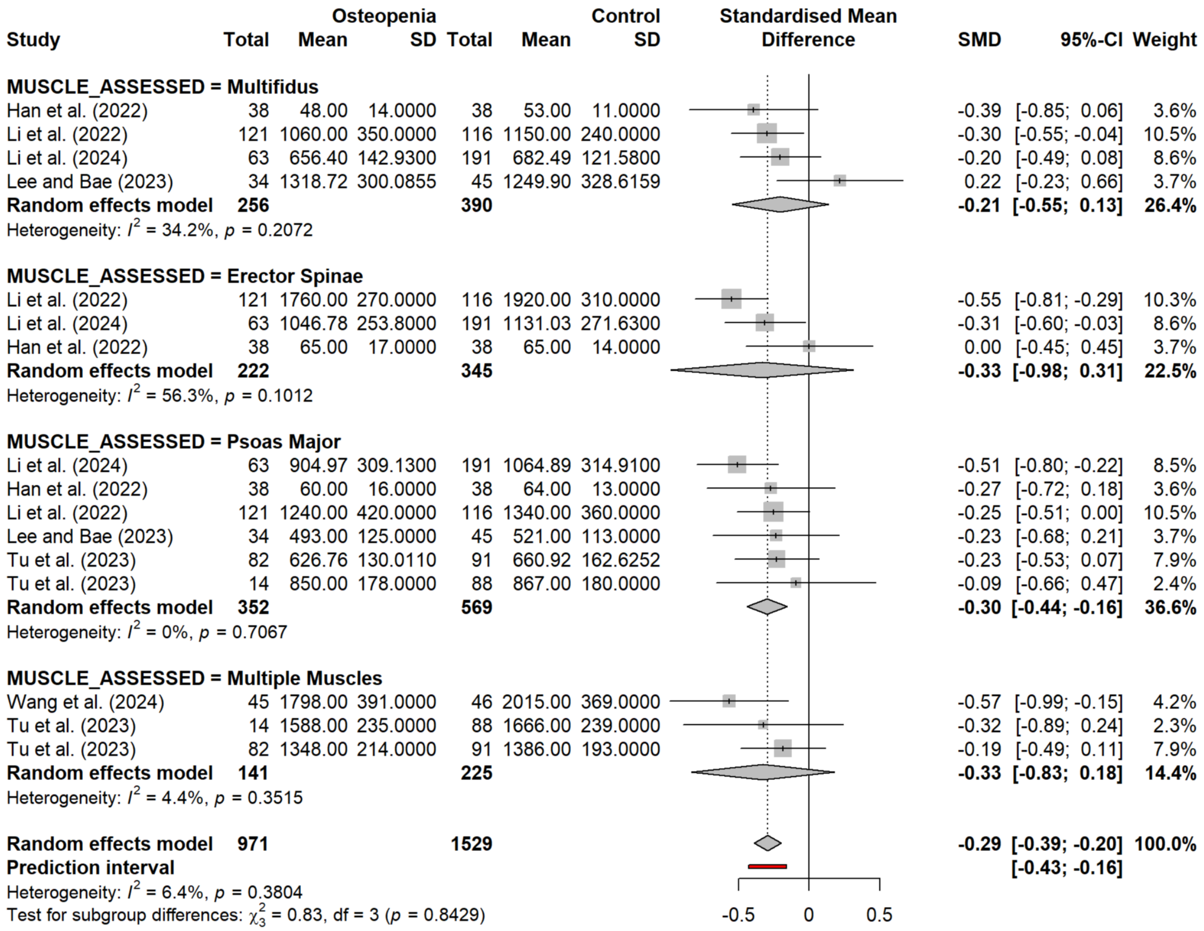

Figure S2: Forest plot of the standardized mean differences in cross-sectional area between individuals with osteopenia and controls, grouped by muscle types.

Figure **S2** showed the forest plot of SMD in CSA between **Osteopenia** patients and group **controls**. Studies were grouped by the muscle assessed, including psoas major, multifidus, erector spinae, and a multiple muscles group that includes data from at least one paraspinal muscle (e.g., multifidus, erector spinae, psoas major, or iliopsoas).

For the multifidus group, the pooled SMD was −0.21 (95% CI: −0.55 to 0.13, $I^2 = 34.2\%$), showing no statistically significant difference. In the erector spinae group, the pooled SMD was −0.33 (95% CI: −0.98 to 0.31, $I^2 = 56.3\%$), also not significant. For the psoas major group, based on six effect sizes from five studies, the pooled result indicated a statistically significant reduction in CSA in osteopenia subjects (SMD = −0.30, 95% CI: −0.44 to −0.16, $I^2 = 0\%$). The Q-test yielded $Q = 2.96$, $p = 0.706$, indicating negligible heterogeneity across included samples. However, given the relatively small number of studies included, the statistical power to detect between-study heterogeneity may be limited, and caution is warranted when interpreting the homogeneity. The multiple muscles group showed a similar trend, with a pooled SMD of −0.33 (95% CI: −0.83 to 0.18, $I^2 = 4.4\%$), though this difference did not reach statistical significance.

The overall random-effects model revealed a small but statistically significant reduction in CSA in the osteopenia group compared to controls (SMD = −0.29, 95% CI: −0.39 to −0.20), with low heterogeneity across studies (I^2 = 6.4%). also, this analysis includes repeated studies assessing different muscles, the overall findings should be further examined using mixed-effects meta-regression models.

Figure S3 Osteoporosis/Osteopenia vs. Control in CSA

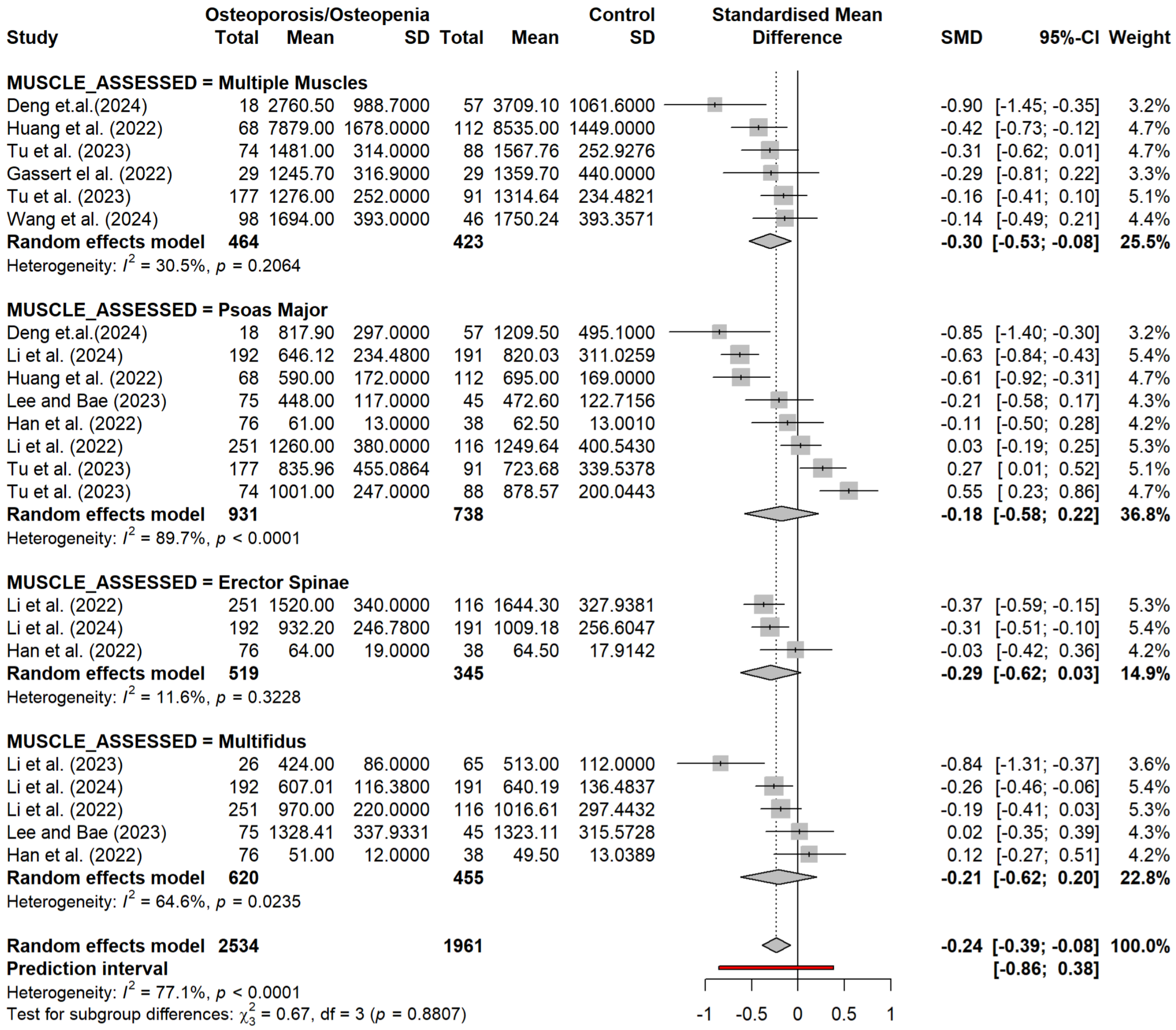

Figure S3: Forest plot of the standardized mean differences in cross-sectional area between individuals with osteoporosis or osteopenia and controls, grouped by muscle types.

Figure **S3** presents the forest plot of SMD in CSA between patients with osteoporosis or osteopenia and control participants. Studies were grouped according to the muscle assessed, including psoas major, multifidus, erector spinae, and a multiple muscles group, which includes data from at least one paraspinal muscle (e.g., multifidus, erector spinae, psoas major, or iliopsoas).

For the multiple muscles group, the pooled SMD was −0.30 (95% CI: −0.53 to −0.08, I^2 = 30.5%), indicating a statistically significant reduction in CSA in the osteoporosis/osteopenia group. In the psoas major group (8 effect sizes from 6 studies), the pooled SMD was −0.18 (95% CI: −0.58 to 0.22, I^2 = 89.7%), suggesting no significant difference and considerable heterogeneity. For the erector spinae group, the pooled SMD was −0.29 (95% CI: −0.62 to 0.03, I^2 = 11.6%), and for the multifidus group, the pooled SMD

was −0.21 (95% CI: −0.62 to 0.20, $I^2 = 64.6\%$), both of which did not reach statistical significance. The overall random-effects model showed a small but statistically significant reduction in CSA in the osteoporosis/osteopenia group compared to controls (SMD = −0.24, 95% CI: −0.39 to −0.08), with moderate heterogeneity across studies ($I^2 = 77.1\%$). also, this analysis includes repeated studies assessing different muscles, the overall findings should be further examined using mixed-effects meta-regression models.

Figure S4 Osteoporosis vs. Osteopenia in CSA

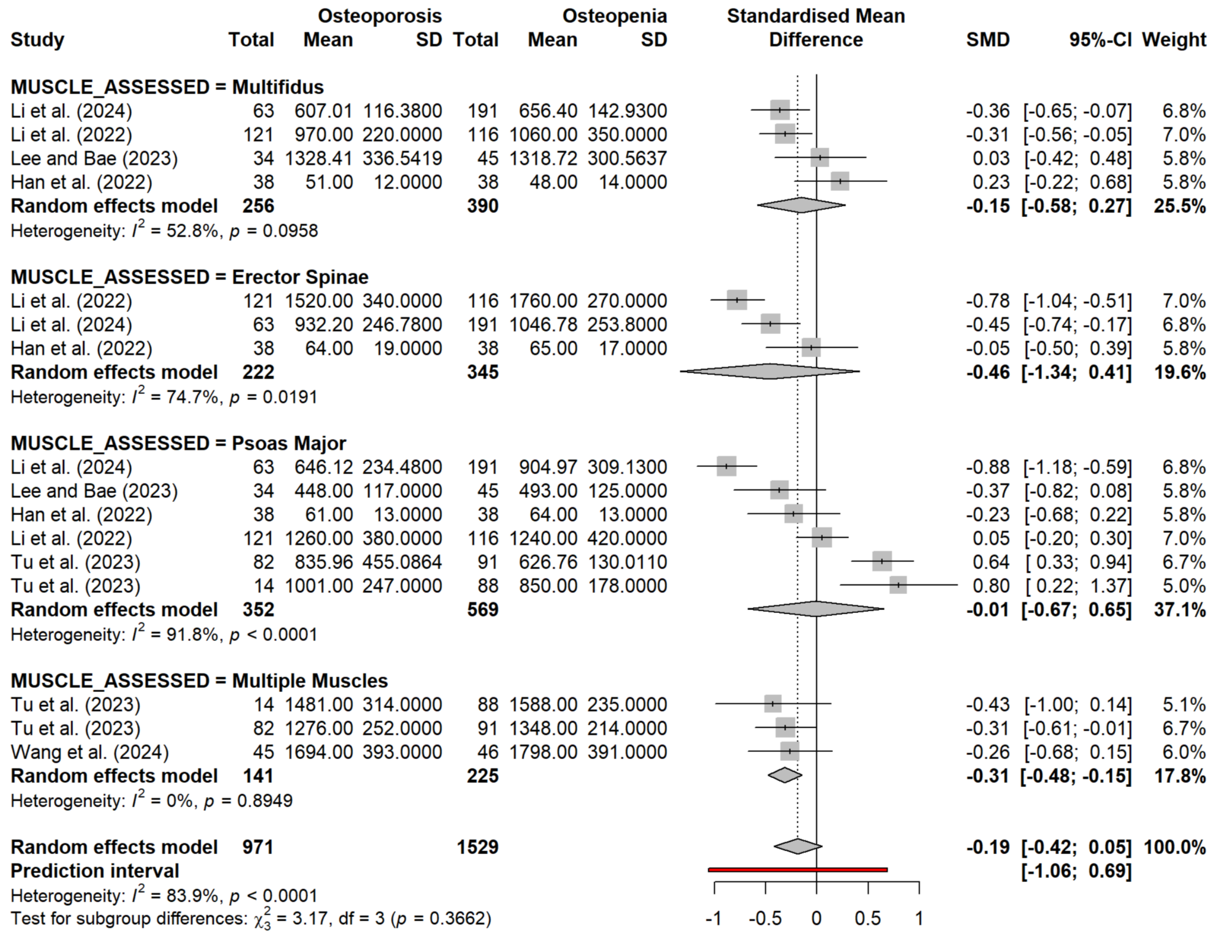

Figure S4: Forest plot of the standardized mean differences in cross-sectional area between individuals with osteoporosis and those with osteopenia, grouped by muscle types.

Figure **S4** illustrates the forest plot of SMD in CSA between individuals with osteoporosis and those with osteopenia. Studies were grouped according to the muscle assessed, including psoas major, multifidus, erector spinae, and a multiple muscles group, which contains data from at least one paraspinal muscle (e.g., multifidus, erector spinae, psoas major, or iliopsoas).

For the multifidus group, the pooled SMD was −0.15 (95% CI: −0.58 to 0.27, $I^2 = 52.8\%$), indicating no statistically significant difference. The erector spinae group yielded a pooled SMD of −0.46 (95% CI: −1.34 to 0.41, $I^2 = 74.7\%$), also not statistically significant. In the psoas major group, based on six effect sizes, the pooled SMD was −0.01 (95% CI: −0.67 to 0.65, $I^2 = 91.8\%$), showing no significant difference and substantial heterogeneity. The multiple muscles group had a pooled SMD of −0.31 (95% CI: −0.48 to −0.15, $I^2 = 0\%$), which reached statistical significance with no observed heterogeneity.

The overall random-effects model suggested a small and non-significant reduction in CSA in the osteoporosis group compared to the osteopenia group (SMD = −0.19, 95% CI: −0.42 to 0.05), with substantial heterogeneity ($I^2 = 83.9\%$). also, this analysis includes repeated studies assessing different muscles, the overall findings should be further examined using mixed-effects meta-regression models.

Figure S5 Osteoporosis vs Non-Osteoporosis in CSA

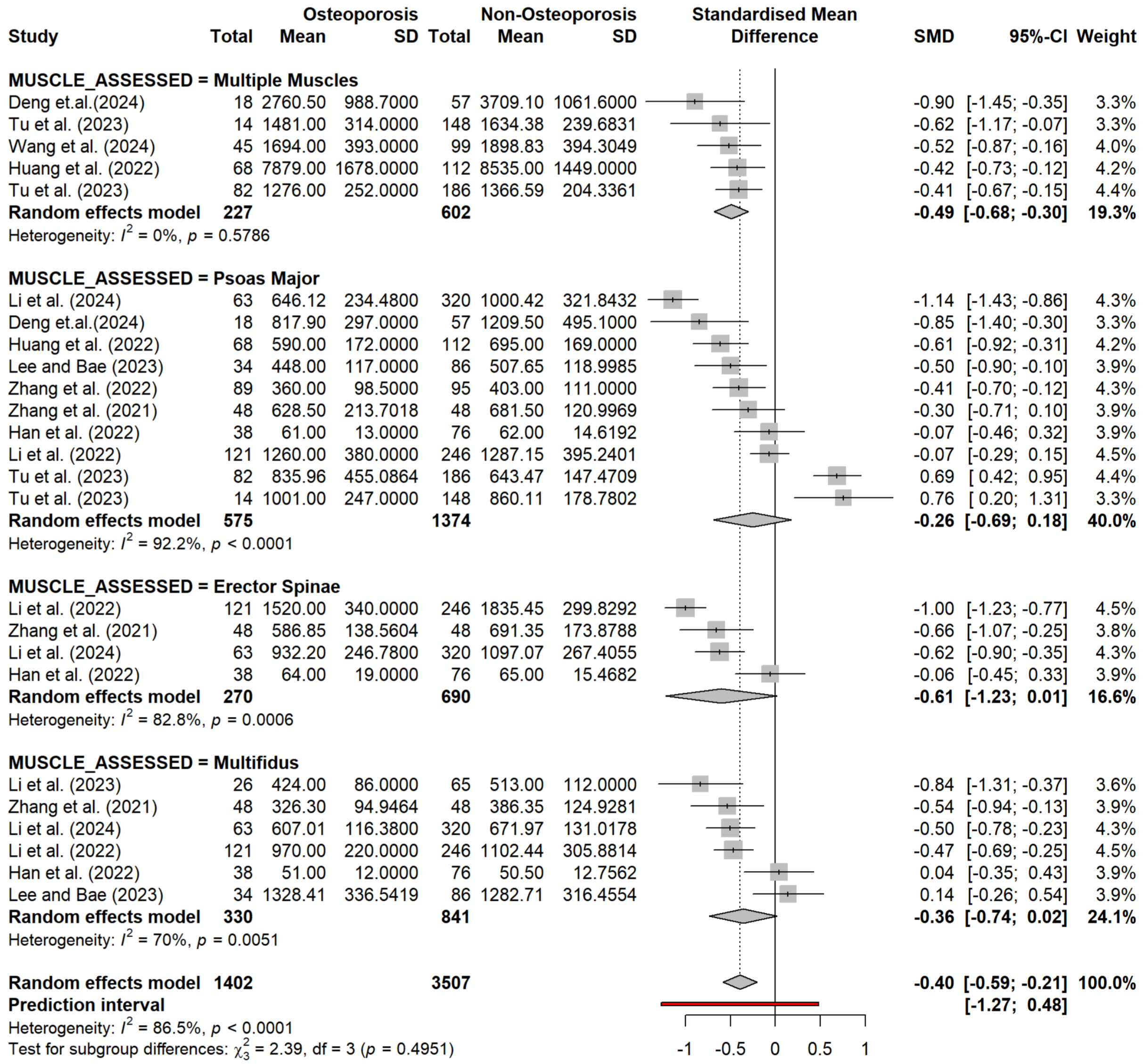

Figure S5: Forest plot of the standardized mean differences in cross-sectional area between individuals with osteoporosis and those without osteoporosis (including both group osteopenia and group control), grouped by muscle types.

Figure **S5** shows the forest plot of SMD in CSA between individuals with osteoporosis and those without osteoporosis (including both osteopenia and control groups). Studies were grouped according to the muscle assessed, including psoas major, multifidus, erector spinae, and a multiple muscles group, which includes data from at least one paraspinal muscle (e.g., multifidus, erector spinae, psoas major, or iliopsoas).

In the multiple muscles group, the pooled SMD was −0.49 (95% CI: −0.68 to −0.30, $I^2 = 0\%$), indicating a significant reduction in CSA in the osteoporosis group. For the psoas major group, the pooled SMD was −0.26 (95% CI: −0.69 to 0.18, $I^2 = 92.2\%$), showing no statistically significant difference and substantial heterogeneity. The erector spinae group showed a pooled SMD of −0.61 (95% CI: −1.23 to −0.01, $I^2 = 82.8\%$), indicating a significant CSA reduction in osteoporosis. In the multifidus group, the pooled SMD was −0.36 (95% CI: −0.74 to 0.02, $I^2 = 70.0\%$), which did not reach statistical significance. The overall random-effects model revealed a moderate and statistically significant reduction in CSA in the osteoporosis group compared to non-osteoporosis individuals (SMD = −0.40, 95% CI: −0.59 to −0.21), with high heterogeneity observed ($I^2 = 86.5\%$). Also, this analysis includes repeated studies assessing different muscles, the overall findings should be further examined using mixed-effects meta-regression models.

Figure S6 Osteoporosis vs. Control in FI

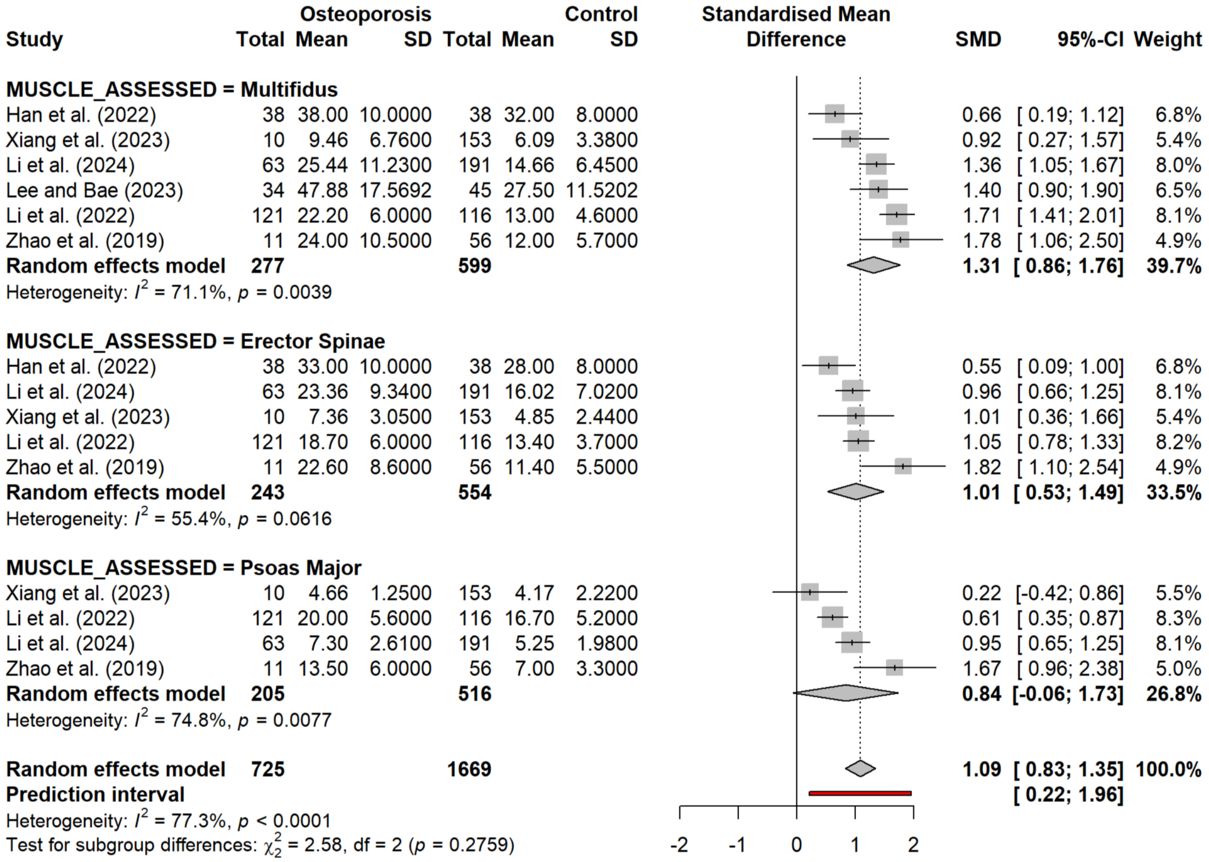

Figure S6: Forest plot of the standardized mean differences in fatty infiltration between individuals with osteoporosis and controls, grouped by muscle type.

Figure **S6** presents the forest plot of SMD in FI between patients with osteoporosis and healthy controls. Due to limited data availability, only three muscle groups were analyzed: multifidus, erector spinae, and psoas major. No combined or multiple-muscle subgroup analysis was performed for FI in this comparison.

For the multifidus, six effect sizes were included from five studies, resulting in a pooled SMD of 1.31 (95% CI: 0.86 to 1.76, $I^2 = 71.1\%$, $p = 0.004$), indicating a significantly higher degree of FI in osteoporosis patients. The erector spinae showed a pooled SMD of 1.01 (95% CI: 0.53 to 1.49, $I^2 = 55.4\%$, $p = 0.062$) based on five effect sizes from five studies. For the psoas major, four effect sizes were included, yielding a pooled SMD of 0.84 (95% CI: −0.06 to 1.73, $I^2 = 74.8\%$, $p = 0.008$). The overall random-effects model combining all muscle

groups indicated a significantly greater FI in the osteoporosis group compared to controls (SMD = 1.09, 95% CI: 0.83 to 1.35), with high overall heterogeneity (I^2 = 77.3%). No significant subgroup differences were detected (Q = 2.58, df = 2, p = 0.276). This analysis includes repeated studies assessing different muscles, the overall findings should be further examined using mixed-effects meta-regression models.

Figure S7 Osteopenia vs. Control in FI

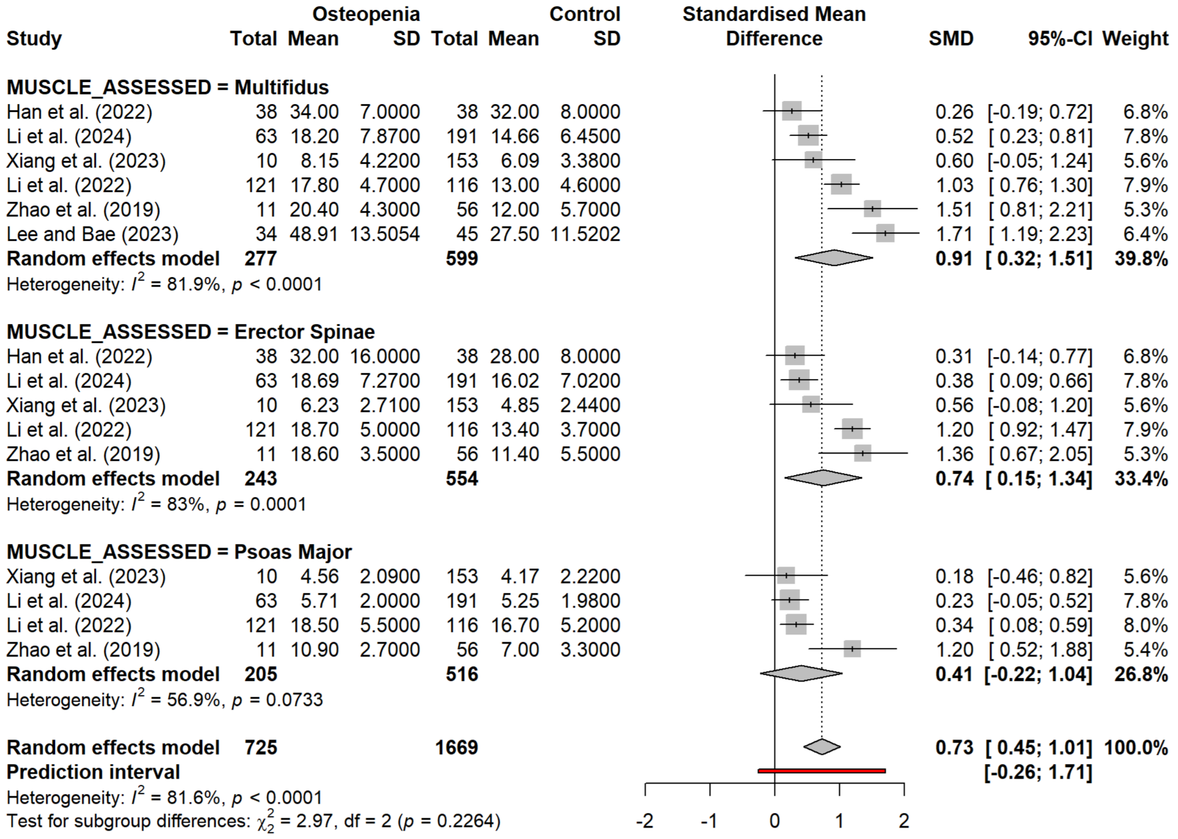

Figure S7: Forest plot of the standardized mean differences in fatty infiltration between individuals with osteopenia and controls, grouped by muscle type.

Figure **S7** displays the forest plot of SMD in FI between individuals with osteopenia and group controls. For the multifidus, six effect sizes were derived from six studies, yielding a pooled SMD of 0.91 with a 95% confidence interval from 0.32 to 1.51 (I^2 = 81.9%, p < 0.001), indicating a significant increase in FI in the osteopenia group. In the erector spinae, five effect sizes were included, resulting in a pooled SMD of 0.74 (95% CI: 0.15 to 1.34, I^2 = 83.0%, p = 0.001). For the psoas major, four effect sizes were included, with a pooled SMD of 0.41 (95% CI: −0.22 to 1.04, I^2 = 56.9%, p = 0.073), and the difference was not statistically significant. The overall random-effects model showed a pooled SMD of 0.73 (95% CI: 0.45 to 1.01), suggesting a higher level of FI in the osteopenia group compared to controls. The overall heterogeneity was considerable (I^2 = 81.6%, p < 0.001). No statistically significant differences were found across muscle subgroups (Q = 2.97, df = 2, p = 0.226). This analysis includes repeated studies assessing different muscles, the overall findings should be further examined using mixed-effects meta-regression models.

Figure S8 Osteoporosis vs. Osteopenia in FI

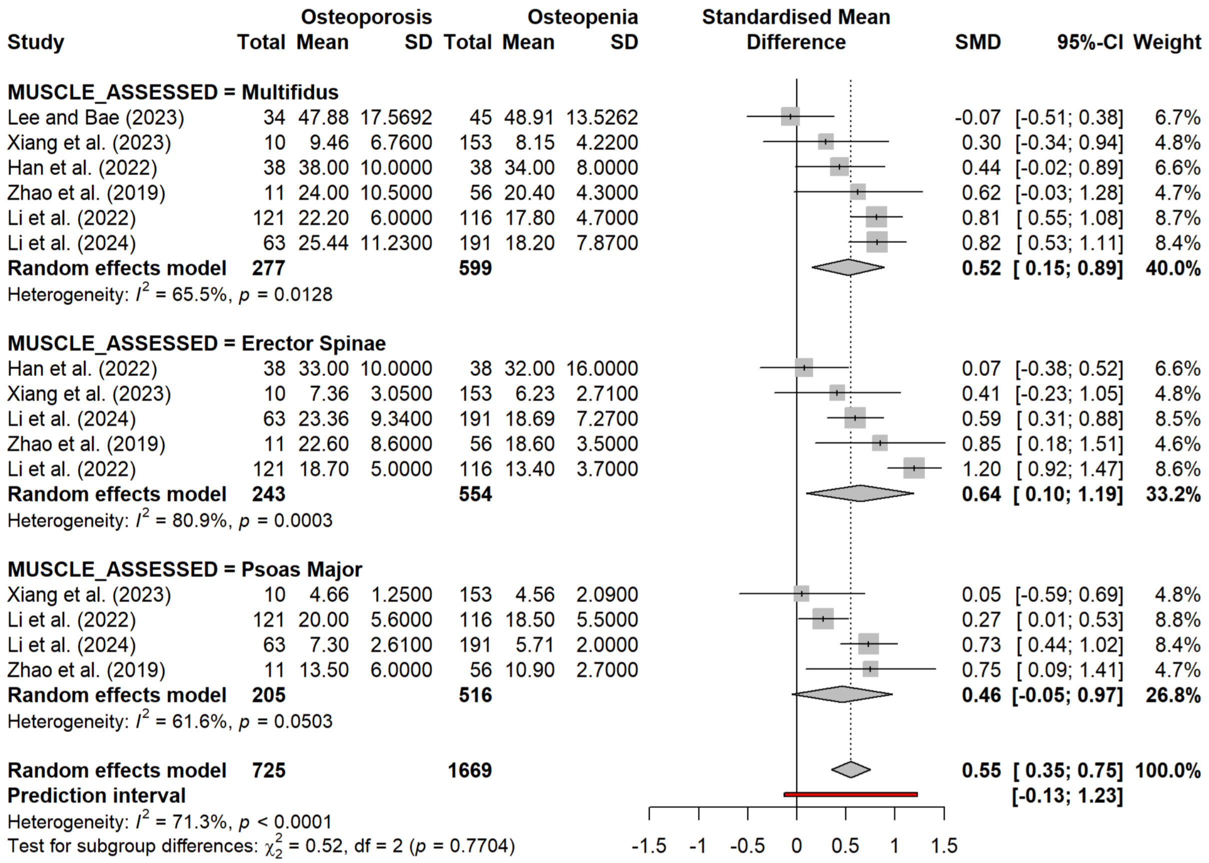

Figure S8: Forest plot of the standardized mean differences in fatty infiltration between individuals with osteoporosis and those with osteopenia, grouped by muscle type.

Figure **S8** presents the forest plot of SMD in FI between individuals with osteoporosis and those with osteopenia. In the multifidus, six effect sizes from six studies were included. The pooled SMD was 0.52 with a 95% confidence interval from 0.15 to 0.89 ($I^2 = 65.5\%$, $p = 0.013$), indicating a significantly greater degree of FI in the osteoporosis group compared to the osteopenia group. For the erector spinae, five effect sizes were analyzed. The pooled SMD was 0.64 (95% CI: 0.10 to 1.19, $I^2 = 80.9\%$, $p = 0.003$), also favoring the osteopenia group. In the psoas major, four effect sizes were included, resulting in a pooled SMD of 0.46 (95% CI: -0.05 to 0.97, $I^2 = 61.6\%$, $p = 0.050$), which did not reach statistical significance. The overall random-effects model indicated a pooled SMD of 0.55 (95% CI: 0.35 to 0.75), with a significant difference in FI between osteoporosis and osteopenia patients. The total heterogeneity was moderate ($I^2 = 71.3\%$, $p < 0.001$). No significant subgroup differences were observed ($Q = 0.52$, $df = 2$, $p = 0.770$). This analysis includes repeated studies assessing different muscles, the overall findings should be further examined using mixed-effects meta-regression models.

Three-Level Meta-Regression Analyses of Study-Level Moderators

Table S4: Results of the three-level meta-regression model with cluster-robust standard errors evaluating the association between effect sizes and study-level moderators.

Moderator	Estimate (95% CI)	SE (df)	t-stat	p-value
Intercept	0.22 (−7.75, 8.20)	3.25 (5.95)	0.07	0.95
Age (year)	−0.03 (−0.09, 0.03)	0.02 (5.69)	−1.40	0.22
Sample Female (%)	0.07 (−0.25, 0.39)	0.12 (4.45)	0.56	0.61
BMI (kg/m^2)	0.01 (−0.31, 0.33)	0.13 (5.75)	0.07	0.95
Outcome Type (*FI*)	0.37 (−0.04, 0.78)	0.15 (3.81)	2.54	0.07
Vertebral Level (*single*)	−0.02 (−0.87, 0.83)	0.35 (6.12)	−0.06	0.95
Muscles (Ref. *Erector Spinae*)				
Multifidus	−0.05 (−0.26, 0.17)	0.08 (4.44)	−0.57	0.60
Psoas Major	−0.11 (−0.62, 0.40)	0.19 (4.66)	−0.55	0.61
Multiple Muscles	0.36 (−0.29, 1.01)	0.19 (2.60)	1.90	0.17

Note: Age represents the mean age of participants in each study. Sample Female (%) denotes the proportion of female participants, calculated per 10% increment. BMI refers to the average body mass index of the sample. Vertebral Level is a binary variable indicating whether muscle measurements were taken at a single vertebral level (coded as "single") or across multiple levels (reference category). The moderator "Outcome Type (FI)" indicates whether the effect size was derived from fatty infiltration (FI) rather than cross-sectional area (CSA), with CSA used as the reference. Muscle group moderators compare *Multifidus*, *Psoas Major*, and *Multiple Muscles* against the reference category.

Table S5: Results of the three-level meta-regression model assessing the influence of diagnostic group comparisons and study-level characteristics on effect sizes.

Moderator	Estimate (95% CI)	SE	z-value	p-value
Intercept	0.19 (−3.63, 4.01)	1.95	0.10	0.92
Age (year)	−0.01 (−0.04, 0.02)	0.01	−0.77	0.44
Sample Female (%)	0.05 (−0.10, 0.21)	0.08	0.64	0.52
BMI (kg/m^2)	0.004 (−0.15, 0.16)	0.08	0.05	0.96
Outcome Type (*FI*)	0.39 (0.22, 0.56)	0.09	4.49	< 0.001
Vertebral Level (*single*)	−0.22 (−0.70, 0.26)	0.24	−0.90	0.37
Muscles (Ref. *Erector Spinae*)				
Multifidus	−0.05 (−0.25, 0.15)	0.10	−0.48	0.63
Psoas Major	−0.13 (−0.33, 0.08)	0.11	−1.22	0.22
Multiple Muscles	0.29 (−0.06, 0.64)	0.18	1.61	0.11
Comparison Category (Ref. *OP vs. OPN*)				
OP vs. Control	0.49 (0.27, 0.70)	0.11	4.50	< 0.001
OPN vs. Control	0.16 (−0.08, 0.41)	0.13	1.30	0.19
OP vs. Non-OP	0.42 (−0.26, 1.10)	0.35	1.20	0.23

Note: Age represents the mean age of participants in each study. Sample Female (%) denotes the proportion of female participants, calculated per 10% increment. BMI refers to the average body mass index of the sample. Vertebral Level is a binary variable indicating whether muscle measurements were taken at a single vertebral level (coded as "single") or across multiple levels (reference category). The moderator "Outcome Type (FI)" indicates whether the effect size was derived from fatty infiltration (FI) rather than cross-sectional area (CSA), with CSA used as the reference. Muscle group moderators compare *Multifidus*, *Psoas Major*, and *Multiple Muscles* against the reference category. Comparison Category indicates the type of diagnostic group contrast used in each effect size calculation. All estimates are based on restricted maximum likelihood estimation (REML).

Assessment of Publication Bias

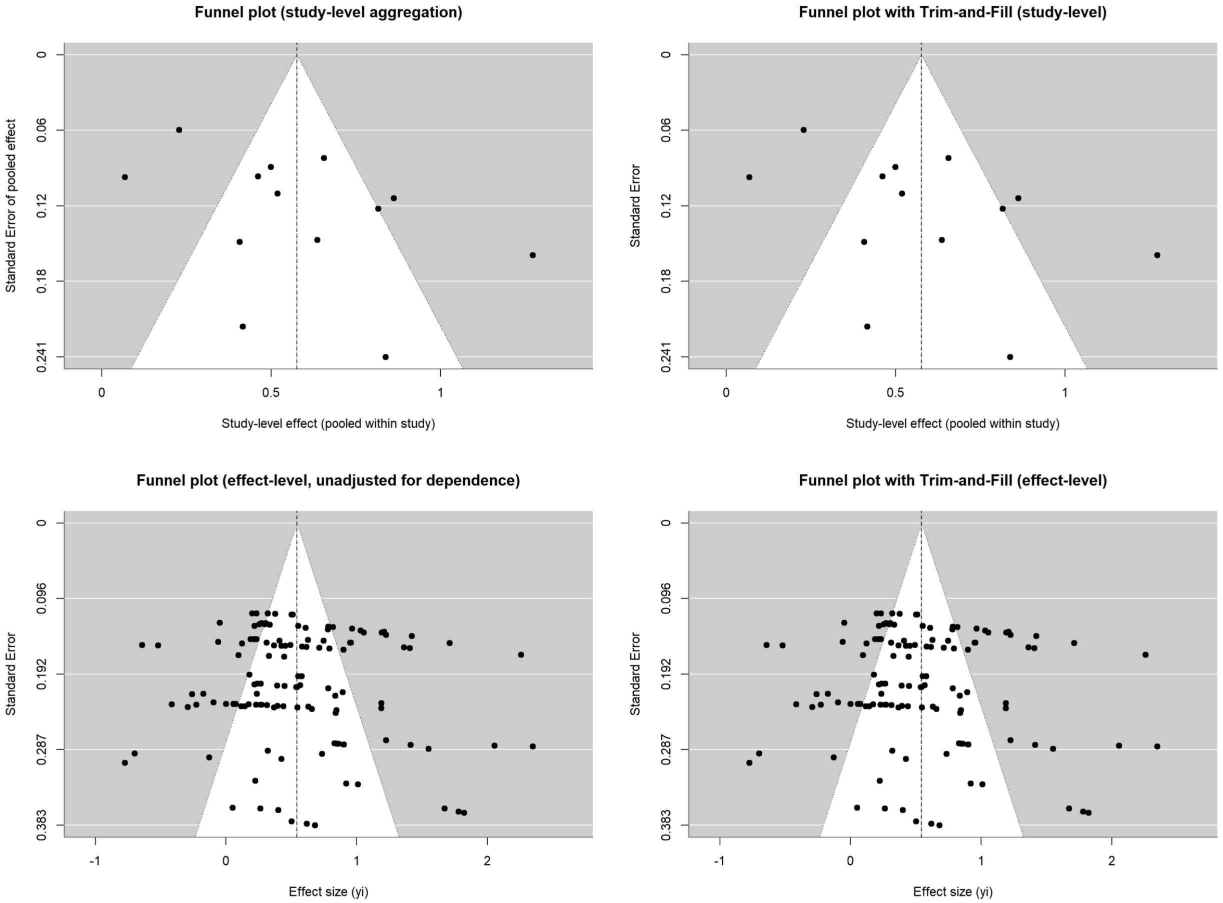

Figure S9: Funnel plots assessing publication bias. (A) Study-level funnel plot. (B) Study-level funnel plot with trim-and-fill. (C) Effect-level funnel plot. (D) Effect-level funnel plot with trim-and-fill. Visual inspection revealed no pronounced asymmetry, and trim-and-fill analyses did not impute additional studies.

Figure S9 presents funnel plots to assess potential publication bias. Study-level funnel plots (Panels A and B) were constructed by pooling multiple outcomes within each study, while effect-level funnel plots (Panels C and D) display all individual outcomes. Visual inspection revealed no pronounced asymmetry at either level. Trim-and-fill analyses did not impute additional studies, further suggesting limited evidence of publication bias.

References

[1] Dongping Deng, Yuxin Shen, and Hongfeng Dong. "Research on the correlation between the area and ADC values of the lumbar and paraspinal muscles measured by MR and osteoporotic vertebral compression fractures". In: *Zhejiang Journal of Traumatology and Surgery* 11 (2024). ISSN 1009-7147, pp. 2026–2029. DOI: `10.39694/issn.1009-7147.2024.11.008`.

[2] Florian Tilman Gassert et al. "Associations between Bone Mineral Density and Longitudinal Changes of Vertebral Bone Marrow and Paraspinal Muscle Composition Assessed Using MR-Based Proton Density Fat Fraction and T2* Maps in Patients with and without Osteoporosis". In: *Diagnostics* 12.10 (Oct. 2022), p. 2467. ISSN: 2075-4418. DOI: `10.3390/diagnostics12102467`.

[3] Gengyu Han et al. "Paraspinal muscle characteristics on MRI in degenerative lumbar spine with normal bone density, osteopenia and osteoporosis: a case-control study". In: *BMC Musculoskeletal Disorders* 23.1 (Jan. 2022). ISSN: 1471-2474. DOI: 10.1186/s12891-022-05036-y.

[4] Cheng-bin Huang et al. "Based on CT at the third lumbar spine level, the skeletal muscle index and psoas muscle index can predict osteoporosis". In: *BMC Musculoskeletal Disorders* 23.1 (Oct. 2022). ISSN: 1471-2474. DOI: 10.1186/s12891-022-05887-5.

[5] Xiangwen Li et al. "Relationship between oseteoporosis with fatty infiltration of paraspinal muscles based on QCT examination". In: *Journal of Bone and Mineral Metabolism* 40.3 (Mar. 2022), pp. 518–527. ISSN: 1435-5604. DOI: 10.1007/s00774-022-01311-z.

[6] Li Dong, Hu Yan-mei, and Zhang Jian-liang. "Predictive value of psoas muscle index for osteoporosis and fracture risk in patients with lumbar degenerative diseases". Chinese. In: *The Journal of Cervicodynia and Lumbodynia* 44.5 (2023), p. 019. DOI: 10.3969/j.issn.1005-7234.2023.05.019.

[7] Ze Li et al. "Relationship between paraspinal muscle properties and bone mineral density based on QCT in patients with lumbar disc herniation". In: *BMC Musculoskeletal Disorders* 25.1 (May 2024). ISSN: 1471-2474. DOI: 10.1186/s12891-024-07484-0.

[8] Song Wang et al. "A Novel MRI-Based Paravertebral Muscle Quality (PVMQ) Score for Evaluating Muscle Quality and Bone Quality: A Comparative Study with the VBQ Score". In: *Clinical Interventions in Aging* Volume 19 (July 2024), pp. 1203–1215. ISSN: 1178-1998. DOI: 10.2147/cia.s464187.

[9] Qingyu Xiang, Xiao Chen, and Guohua Wang. "A correlative study among bone mineral density of lumbar vertebrae, fat content of paravertebral muscle and liver in middle-aged and young people". Chinese. In: *J Pract Radiol* 42.2 (2023), pp. 223–227. DOI: 10.3969/j.issn.1002-1671.2023.02.024.

[10] Yinxia Zhao et al. "Fatty infiltration of paraspinal muscles is associated with bone mineral density of the lumbar spine". In: *Archives of Osteoporosis* 14.1 (Oct. 2019). ISSN: 1862-3514. DOI: 10.1007/s11657-019-0639-5.

[11] Yun Tu et al. "A preliminary study on degenerate characteristics of lumbar and abdominal muscles in middle-aged and elderly people with varying bone mass". In: *BMC Musculoskeletal Disorders* 24.1 (Feb. 2023). ISSN: 1471-2474. DOI: 10.1186/s12891-023-06229-9.

[12] Yihui Zhang et al. "Correlation of Psoas Muscle Index with Fragility Vertebral Fracture: A Retrospective Cross-Sectional Study of Middle-Aged and Elderly Women". In: *International Journal of Endocrinology* 2022 (Nov. 2022). Ed. by Christian-Heinz Anderwald, pp. 1–7. ISSN: 1687-8337. DOI: 10.1155/2022/4149468.

[13] Zhang Wei et al. "Lumbar paraspinal muscle quantity and quality in postmenopausal women with osteoporotic vertebral compression fractures: a case-control study based on MRI". Chinese. In: *Orthopaedics* 22.1 (2021), pp. 1–6. DOI: 10.3969/j.issn.1674-8573.2021.01.002.

[14] Dong Gyu Lee and Jae Hwa Bae. "Fatty infiltration of the multifidus muscle independently increases osteoporotic vertebral compression fracture risk". In: *BMC Musculoskeletal Disorders* 24.1 (June 2023). ISSN: 1471-2474. DOI: 10.1186/s12891-023-06640-2.

[15] Joanna Briggs Institute. *JBI Critical Appraisal Checklist for Cohort Studies.* The Joanna Briggs Institute. Adelaide, Australia, 2020.

[16] Joanna Briggs Institute. *Checklist for Case Control Studies.* JBI Critical Appraisal Tools. Adelaide, 2017.

[17] Joanna Briggs Institute. *Checklist for Analytical Cross Sectional Studies.* JBI Critical Appraisal Tools. Adelaide, 2017.
